# Targeted Delivery of Doxorubicin-Loaded Poly (ε-caprolactone)-b-Poly (N-vinylpyrrolidone) Micelles Enhances Antitumor Effect in Lymphoma

**DOI:** 10.1371/journal.pone.0094309

**Published:** 2014-04-08

**Authors:** Sumit Kumar Hira, Avnish Kumar Mishra, Biswajit Ray, Partha Pratim Manna

**Affiliations:** 1 Immunobiology Laboratory, Department of Zoology, Faculty of Science, Banaras Hindu University, Varanasi, India; 2 Department of Chemistry, Faculty of Science, Banaras Hindu University, Varanasi, India; RMIT University, Australia

## Abstract

**Background:**

The present study was motivated by the need to design a safe nano-carrier for the delivery of doxorubicin which could be tolerant to normal cells. PCL_63_-b-PNVP_90_ was loaded with doxorubicin (6 mg/ml), and with 49.8% drug loading efficiency; it offers a unique platform providing selective immune responses against lymphoma.

**Methods:**

In this study, we have used micelles of amphiphilic PCL_63_-b-PNVP_90_ block copolymer as nano-carrier for controlled release of doxorubicin (DOX). DOX is physically entrapped and stabilized in the hydrophobic cores of the micelles and biological roles of these micelles were evaluated in lymphoma.

**Results:**

DOX loaded PCL_63_-b-PNVP_90_ block copolymer micelles (DOX-PCL_63_-b-PNVP_90_) shows enhanced growth inhibition and cytotoxicity against human (K-562, JE6.1 and Raji) and mice lymphoma cells (Dalton's lymphoma, DL). DOX-PCL_63_-b-PNVP_90_ demonstrates higher levels of tumoricidal effect against DOX-resistant tumor cells compared to free DOX. DOX-PCL_63_-b-PNVP_90_ demonstrated effective drug loading and a pH-responsive drug release character besides exhibiting sustained drug release performance in in-vitro and intracellular drug release experiments.

**Conclusion:**

Unlike free DOX, DOX-PCL_63_-b-PNVP_90_ does not show cytotoxicity against normal cells. DOX-PCL_63_-b-PNVP_90_ prolonged the survival of tumor (DL) bearing mice by enhancing the apoptosis of the tumor cells in targeted organs like liver and spleen.

## Introduction

Adriamycin or Doxorubicin (DOX) hydrochloride, an anthracycline antibiotics is considered as the most effective chemotherapeutics used for the treatment of cancers including acute lymphoblastic and myelogenous leukemia, sarcomas, pediatric solid tumors, non-Hodgkin's and Hodgkin's lymphoma, neuroblastoma, and carcinomas of breast, ovaries and thyroid. The cytotoxic effects of DOX include DNA double helix intercalation, inhibition of topoisomerase II, production of reactive oxygen species (ROS), mitochondrial dysfunction, induction of p53, and activation of caspases [1], [2]. However, its short biological life span, nonspecific distribution, development of drug resistance and severe cardiac toxicity including development of cardiomyopathy, have restricted its success [3]. Several polymer based delivery systems like polymeric micelles [4], synthetic polymer conjugates [5], and antibody targeted carriers [6] have been designed to reduce or alter toxicity in organs like heart, and enhances its potential to the site of drug actions like tumors. Yet the therapeutic efficacy of these formulations has not been demonstrated although modest increase has been reported in certain cases. The versatility of Poly-(ε-caprolactone) (PCL) as a model polymer for pharmaceutical formulations along with functionalization features have been demonstrated over the past decade justifies its immense usefulness [7]. PCL modifications could provide better flexibility including modifications in drug release pattern, micellar drug delivery, tissue compatibility and circumvention of multi drug resistance [8]. Amphiphilic block copolymers have both hydrophobic and hydrophilic segments and undergoes self-assembly, which give rise to its typical aqueous solution and dispersion properties.

Amphiphilic block copolymers containing hydrophilic poly (N-vinylpyrrolidone) (PNVP) segment have several biologically important criteria's including high water solubility, low toxicity, biocompatibility, complexation capability, cryo-protectivity, lypoprotectivity and anti biofouling properties. Very few reports of the synthesis and characterization of amphiphilic block copolymers containing a biocompatible hydrophilic poly (N-vinylpyrrolidone) (PNVP) block and a biodegradable and biocompatible hydrophobic poly (ε-caprolactone) (PCL) block, prepared via conventional radical polymerization of N-vinylpyrrolidone (NVP) are available in the literature [9]–[11]. Recently, Jeon et al. have reported the synthesis and characterization of well-defined amphiphilic PNVP-b-PCL block copolymers prepared through the combination of cobalt-mediated controlled radical polymerization of NVP and controlled ROP of CL [12]. We have recently reported the synthesis of well-defined amphiphilic block copolymers of CL and NVP by combining the controlled ROP of CL and the controlled metal-free xanthate-mediated RAFT polymerization of NVP. Self-assembly behavior of the obtained amphiphilic block copolymers was studied in details using ^1^H NMR, TEM, fluorescence spectroscopy, and light scattering [13].

Herein, we report the synthesis of a PCL-based amphiphilic polymeric nano delivery system DOX-PCL_63_-b-PNVP_90_, which is highly efficient in delivering DOX to tumor targets and also showed enhanced performance in DOX resistant forms as well. DOX-PCL_63_-b-PNVP_90_ is significantly less toxic compared to free DOX against various cell subsets including lymphocytes, which are specifically susceptible to doxorubicin mediated death. DOX-PCL_63_-b-PNVP_90_ prolonged the survival of tumor bearing mice compared to free DOX and restricts the metastasis of lymphoma to other organs. Besides that, DOX-PCL_63_-b-PNVP_90_ prevents the accumulation of doxorubicin in targeted organs like heart compared to free DOX, suggesting its unique suitability for therapeutic purposes against lymphoma.

## Materials and Methods

### Reagents

Triethylamine (TEA) (Loba Chemie, Mumbai, India, 99%), 2-bromopropionyl bromide (Fluka, Israel, >97%), stannous 2-ethylhexanoate [Sn(Oct)_2_] (Aldrich, St Louis, USA, 99%), diethyl ether (s.d.fine, Mumbai, India), hexane (CDH, Mumbai, India), methanol (Loba Chemie, Mumbai, India, 99%), sodium hydrogen carbonate (Loba Chemie, Mumbai, India), ammonium chloride (s.d.fine, Mumbai, India), anhydrous magnesium sulfate (Loba Chemie, Mumbai, India) were used as received. Benzyl alcohol (s.d.fine, Mumbai, India, 99%) was dried over CaO and then distilled under reduced pressure. *ε*-Caprolactone (CL) (Aldrich, St Louis, USA, 99%) was dried over calcium hydride (CaH_2_) for 48 h at room temperature and then distilled under reduced pressure before use. *N*-Vinylpyrrolidone (Aldrich, St Louis, USA, 99%) was dried over anhydrous magnesium sulfate and distilled under reduced pressure. 2, 2^/^- Azobis (isobutyronitrile) (AIBN) (Spectrochem, Mumbai, India, 98%) was recrystallized from methanol. Tetrahydrofuran (THF) (Loba Chemie, Mumbai, India) was dried and fractionally distilled from sodium and benzophenone. Ethanol (Saraya Distilliary, India) was stirred over CaO overnight and distilled over fresh CaO. Potassium *O*-ethyl xantate was prepared according to our previous work [14]. PCL_63_-*b*-PNVP_90_ was synthesized according to our recently published method [13]. Doxorubicin (Adriamycin) was purchased from Selleckchem (S1208) South Loop West, Suite 225, Houston, TX 77054, USA. RPMI 1640, penicillin and streptomycin were purchased from GIBCO, Invitrogen, Carlsbad, CA and fetal bovine serum from Hyclone, Logan, UT. Hoechst 33342 was purchased from Himedia, India. Annexin V Apoptosis Detection Kit (sc-4252 AK) was purchased from Santa Cruz Biotechnology, Dallas, TX, U.S.A. Other general & fine chemicals unless otherwise stated were purchased from SIGMA-ALDRICH, St. Louis, MO, USA.

### Cell Lines and Cell Culture

Dalton lymphoma (DL) was maintained in the peritoneum of AKR (H2k) mice by periodic transfer of tumor cells *via* intraperitoneal injection [15], [16]. Human erytholeukemic cell line K-562, T cell leukemia line JE6.1, and Burkitt lymphoma cell line Raji were kind gift of Dr. Santu Bandyopadhyay, IICB, Kolkata. The cell lines were originally obtained from American Type Culture Collection (ATCC), Manahass, USA. The cells were maintained in RPMI 1640 (Invitrogen, Carlsbad, CA), supplemented with 10% fetal bovine serum (Hyclone, Logan, UT), 100 U/ml penicillin and 100 μg/ml streptomycin (Invitrogen, Carlsbad, CA), henceforth, called as complete medium. The cell lines used in the study were free from mycoplasma. To generate DOX-resistant (DOX-R) cell lines like K-562/DOX-R, JE6.1/DOX-R, Raji/DOX-R, and DL/DOX-R, parental K-562, JE6.1, Raji, and DL cells were cultured in complete medium, with increasing concentrations of DOX up to a final concentration of 1 μM at 5% CO_2_, 37°C for one month. Resistant Cells were subsequently cloned by limiting dilution and grown in complete medium. The established cell lines were maintained in complete medium supplemented with 200 nM DOX to maintain the resistant phenotype. All DOX resistance cells were cultured in the DOX free medium for 14 days prior to experiments.

### Preparation of DOX-PCL_63_-b-PNVP_90_

DOX-loaded polymeric micelles were prepared by dialysis method. In the dialysis method, the copolymer PCL_63_-*b*-PNVP_90_ (20 mg) was dissolved in DMF (2 ml), and a corresponding DOX·HCl (6 mg) with TEA (3 mol eq. to DOX·HCl) were added into the polymer solution. The mixture was stirred at room temperature for 24 h. Then, the final mixture was dialyzed against distilled water using a dialysis membrane [molecular weight cut off (MWCO) = 3500 g mol^−1^] for 8 h. During the first 3 h, the water was exchanged three times (every hour) and then twice during the following 5 h. The dialyzed solution was finally concentrated to 2 mL followed by lyophilisation to yield the solid micelle sample. Morphology and size of the micelles were determined by Transmission Electron Microscopy (TEM) (JOEL, JEM, 2100) operated at an acceleration voltage of 120 kV. The TEM samples were prepared by putting a drop of aqueous block copolymer solution (1 mg/mL) on the carbon coated copper grid followed by the removal of extra solution with a filter paper. The Dynamic Light Scattering Instrument (Malvern Zetasizer Ver. 7.01 Serial Number: MAL1077742) was performed to study the hydrodynamic size (Rh) of the free micelles and DOX-PCL_63_-*b*-PNVP_90_ micelles using 0.5 mg/ml solution at 90^o^ angle.

### Determination of Drug-loading Content (DLC) and Drug-loading Efficiency (DLE) for DOX

The DLC was considered as the weight percentage of DOX in the micelle. It was quantified by determining the absorbance at 451 nm using a UV–Vis spectrophotometer (Shimadzu UV-1700). 1 mg of lyophilized sample was dissolved in 2 mL DMF for the UV–Vis measurement. To generate a calibration curve for the DLC calculation of DOX-loaded micelle, DOX solutions of various concentrations were prepared, and the absorbance at 485 nm was measured. DLC and DLE are calculated using the following two formula respectively:
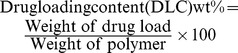
, 
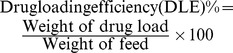
.

### Typical Drug Release from DOX-PCL_63_-b-PNVP_90_ Micelles

5 mg of lyophilized DOX-loaded polymeric micelle dissolved in 1 ml phosphate buffer saline (PBS) of 6.4/7.4 pH was taken in a dialysis bag with a MWCO of 3500 g mol^−1^, which was placed into 20 ml PBS solution. At different intervals, 3.0 ml was removed from the outer aqueous solution and replaced by fresh release medium (PBS solution). The released drug was quantified spectrophotometrically at 483 nm. The test was performed at 37°C.

### In-vitro Tumor Cell Growth Inhibition Assay

Growth inhibitory potential of free DOX, PCL_63_-*b*-PNVP_90_ micelle and DOX-PCL_63_-*b*-PNVP_90_ micelle against parental and DOX-resistant DL, K-562, JE6.1, and Raji cells were studied by MTT assay. In a 96-well tissue culture plate 5×10^3^ cells/well were added and exposed to free DOX, PCL_63_-*b*-PNVP_90_ micelle or, DOX-PCL_63_-*b*-PNVP_90_ micelle solution with serial concentrations of (0.0001, 0.005, 0.01, 0.05, 0.1, 0.5, 1, 5 μM). Plates were incubated at 37°C, 5% CO_2_, for 48 h. The cell proliferation was measured by CellTiter 96 Non-Radioactive Cell Proliferation Assay (MTT) kit from Promega, USA according to the manufacturer's protocol. The plates were incubated for 4 h with the MTT reagent and absorbance was measured at 570 nm using Synergy HT Multi-Mode Micro plate Reader BioTek, USA. The data presented as the percentage of inhibition of tumor cells and was calculated from the following formula: 
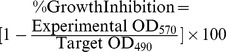
 Where Experimental OD value is the reading of tumor cells exposed to various concentrations of DOX and Target OD value is the corresponding value of tumor cell only cultured in the absence of drug.

### DOX-PCL_63_-b-PNVP_90_ Cytotoxicity Assay

The lytic activity of DOX-loaded PCL_63_-*b*-PNVP_90_ was measured by 18 h non-radioactive cytotoxicity assay using the CytoTox 96 Non-Radioactive Cytotoxicity assay kit from Promega, USA, which quantitatively measures lactate dehydrogenase (LDH), a stable cytosolic enzyme released upon cell lysis. Target cells (5×10^3^) were added to 96-well tissue culture plate and exposed to serial concentrations (0.0001, 0.0002, 0.0005, 0.001, 0.005, 0.01, 0.05 μM) free DOX, PCL_63_-*b*-PNVP_90_ micelle or, DOX-PCL_63_-*b*-PNVP_90_ micelle solution and incubated for 18 h at 37°C, 5% CO_2_. After incubation, the released LDH in culture supernatants was measured with a 30-minute coupled enzymatic assay, which results in the conversion of a tetrazolium salt (INT) into a red formazan product. The amount of color formed is proportional to the number of lysed cells. Visible wavelength absorbance data at 490 nm were collected using a standard 96-well plate reader. Percent-specific lysis was determined using the following formula: 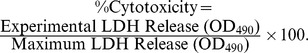.

### Clonogenic Survival Assay

The clonogenic survival assay was performed according to the method described by Nicolaas A P Franken *et al.* [17] with some modifications. Cells (10 μL from a stock (1×10^4^ cells/ml) were treated with 0.1 to 5.0 μM of the DOX-PCL_63_-*b*-PNVP_90_ or, free DOX for 24 h. Cells treated with free micelles were used as control. The cells were washed, diluted in RPMI containing 10% (v/v) fetal calf serum, the supplements listed above, and 0.3% noble agar (Difco, Detroit, MI) and plated in 6-well plate in triplicate on soft agar. Once set, the dishes were overlaid with 1.0 ml complete medium and incubated at 37°C. After 10 days, the total number of colonies/plate was counted. Plating efficiency (PE) and surviving fraction (SF) were calculated as mean ± SD of triplicate by following formula: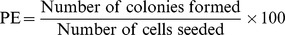, The number of colonies that arise after treatment of cells, expressed in terms of PE, is called the surviving fraction (SF): 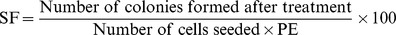.

### Effect of DOX-PCL_63_-b-PNVP_90_ Micelles on Viability of Human Lymphocytes, Monocytes and Dendritic Cell

Effect of free DOX or, DOX-PCL_63_-*b*-PNVP_90_ micelles on the proliferation of human lymphocytes, monocytes or dendritic cells (DC) was evaluated by XTT assay. Lymphocytes were collected from peripheral blood by differential centrifugation in Ficoll-Hypaque. Monocytes were isolated by adhering total PBMC by glass adherence. Dendritic cells were generated by incubating monocytes with recombinant GM-CSF and IL-4 for 7 days as described elsewhere. Lymphocytes, monocytes or DC were plated (5×10^3^ cells/well) in a 96-well plate and exposed to serial concentrations of (0.0001, 0.005, 0.01, 0.05, 0.1, 0.5, and 1 μM) free DOX or, DOX-PCL_63_-*b*-PNVP_90_ micelle and incubated at 37°C, 5% CO_2_, for 48 h. The cell viability was measured by XTT cell viability assay kit from Cell Signaling TECHNOLOGY, USA according to the manufacturer's protocol. OD was measured at 450 nm using Synergy HT Multi-Mode Micro plate Reader BioTek, USA. The data was presented as the percentage of viable cell calculated from the following formula: 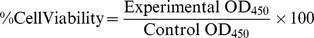.

### Quantification of DOX-PCL_63_-*b*-PNVP_90_ Conjugate in Cells using Fluorescence Plate Reader

The intracellular concentration of the DOX-PCL_63_-*b*-PNVP_90_ was determined by detecting intracellular fluorescence intensity of DOX. Briefly, both parental and DOX-resistant K-562, JE6.1, Raji, DL and human DC, lymphocytes were plated in 96-well culture plates at concentration of 2×10^4^ cells/well. Plated cells were treated with the DOX-PCL_63_-*b*-PNVP_90_ micelles or, free DOX at a concentration of 2.0 μM. Cells treated with free micelles acts as control. After incubation at 37°C for 0.5, 2, 4, 8, 16, and 24 h, each culture medium was removed and cells were washed three times with cold PBS. The cells were lysed in 200 μL of a lysis buffer (50 mM Tris-HCL, 150 mM NaCl, 0.1% NP40, 0.1% SDS, 0.1% sodium deoxycholate, 1% Triton X-100). For fluorimetric analysis, total cellular DOX in Dox-PCL_63_-*b*-PNVP_90_ micelles was directly determined by measuring the fluorescent emission of the solution (λ_ex_ = 480 nm, λ_em_ = 590 nm) in the cell lysate with Synergy HT Multi-Mode Micro plate Reader BioTek, USA. The conjugate concentration was given by the standard curve of DOX.

### Fluorescence Microscopy for Doxorubicin Uptake

Parental or doxorubicin resistant K-562 and DL cells were plated in a 24 well plates at concentration of 5×10^3^ cells per well. Cells were washed twice with PBS and incubated with free doxorubicin or DOX-PCL_63_-b-PNVP_90_ micelles with equivalent concentration of doxorubicin (5.0 μM) in complete medium for 4 h at 37°C. Cells were washed twice with ice-cold PBS and fixed with freshly prepared 2% paraformaldehyde for 15 minutes at room temperature. The cells were counterstained with Hoechst stain (for nucleus staining) and mounted on glass microscope slides with a drop of mounting media to reduce fluorescence photo bleaching. The intracellular DOX localization was visualized under a fluorescence microscope Eclipse 80i (Nikon, Japan) (Plan Fluor, 40X, NA 0.75 objective) and images were acquired by DS-Fi 1c CCD camera using imaging software NIS-Elements F 3.2 (Nikon, Japan) at 22°C. Image analysis, merging was performed by using Image-Pro Plus AMS analysis software (Media Cybernetics, Inc. 401 N. Washington Street, Suite 350 Rockville, MD 20850 USA).

### Flow Cytometry Analysis for Doxorubicin Uptake

Parental or DOX-resistant (DOX/R) K-562, JE6.1, Raji, and DL cells were plated in 24-well plates at a concentration of 5 × 10^3^ cells per well. Cells were washed twice in PBS, and incubated with free DOX or, DOX-PCL_63_-*b*-PNVP_90_ micelles with equivalent doxorubicin at a concentration of 5.0 μM, in complete RPMI 1640 medium for 4 h at 37°C. Following incubation, the cells were washed twice with PBS and then re-suspended in 500 μl PBS. Fluorescence histograms were then recorded with a BD FACS Calibur flow cytometer (Beckton Dickinson, U.S.A.) in FL3 channel. We analyzed 20,000 events to generate each histogram.

### Intracellular DOX Release and Efflux Study

For the time course study, K-562, JE6.1, Raji, and DL cells were incubated with 5.0 μM DOX-PCL_63_-*b*-PNVP_90_ micelles for 12 h. To examine the efflux process, the culture medium containing 5.0 μM DOX-PCL_63_-*b*-PNVP_90_ micelles was replaced by a DOX free medium after 12 h incubation. Cells were harvested at 2, 4, 8, 16, and 24 h later, respectively. The fluorescence of DOX-PCL_63_-*b*-PNVP_90_ micelles in cells was measured using fluorescence plate reader. The determination process was described as above. The conjugate concentration was given by the standard curve.

### Detection of Apoptosis

Evaluation of apoptotic cell death in parental and doxorubicin resistant (DOX/R) K-562, JE6.1, Raji or DL by free doxorubicin or the doxorubicin conjugated PCL_63_-*b*-PNVP_90_ micelles was assessed by binding FITC-conjugated Annexin V. After 18 h of incubation, the percentages of FITC-conjugated Annexin V-positive cells were analyzed by BD FACS Calibur flow cytometer (Beckton Dickinson, U.S.A.). In some experiments after apoptosis induction, cells were washed twice with ice-cold PBS and stained with FITC-conjugated Annexin V for 20 minutes. These cells were washed in Annexin buffer and were mounted on microscope slides with a drop of mounting media to reduce fluorescence photo bleaching. The FITC-conjugated Annexin V positive cell & intracellular DOX localization was visualized under a fluorescence microscope (Nikon Eclipse 80i, Nikon, Japan) as describe earlier.

### Murine Lymphoma Model

AKR/J mice were maintained and bred under pathogen-free condition of the central animal house facility of the department. Use of mice was approved by the Institutional Animal Ethics Committee, Banaras Hindu University. All animal experiments were performed according to the National Regulatory Guidelines issued by Committee for the Purpose of Supervision of Experiments on Animals (CPSEA), Ministry of Environment and Forest, Government of India. The animals were euthanized by cervical dislocation to reduce the suffering as minimum as possible and was performed according to AVMA Guidelines on Euthanasia (AVMA 2007). Tumors (DL) in mice were maintained by transplanting fresh tumor cells in PBS (3×10^4^ cells/mouse) intraperitoneally. All tumor measurements were performed in a blinded fashion. Mice (n = 15/group) were transplanted with tumor and after 96 h (Day 0) were treated with the DOX-PCL_63_-*b*-PNVP_90_ micelles or, free doxorubicin in PBS and were administered intraperitoneally (3 mg kg^-1^ body weight). Mice treated with free micelles were used as control. Altogether 9 doses were given in two phases which includes 4 doses (from day 0 to day 4) given every day and remaining 5 booster doses were given from day 10 to day 20 at an interval of 48 h, respectively. The formulation was prepared and validated such that 100 μl of DOX-PCL_63_-b-PNVP_90_ micelles contained 3 mg kg^−1^ body weight of DOX. PBS (100 μl) was used as vehicle control for drug treatment. The tumor volumes (abdominal circumference) and body weights were monitored on a daily basis. The animals (n = 3) were sacrificed when the average abdominal circumference of the control (PBS only) exceeded 15.5 cm. Before sacrifice, tumor cells were collected in the form of ascitic fluid from peritoneum and blood by retro orbital bleeding. Blood films were prepared, fixed (methanol) and stained with Leishman stain to study the effects of DOX on leukocytes. Organs (spleen, liver, heart, lung, and kidney) were dissected out & weighed. Organs were cut into three parts. One portion was preserved in 10% formalin for further analysis and the other two parts were weighed again for DOX distribution and flow cytometry studies. Mice (n = 12/group) were under observation for 50 days when final data collection was made for Kaplan Mayer survival analysis.

### Doxorubicin Distribution in Various Organs after Therapy

The tissue blocks (1 mg) were homogenized in lysis buffer solution (50 mM Tris-HCL, 150 mM NaCl, 0.1% NP40, 0.1% SDS, 0.1% sodium deoxycholate, 1% Triton X-100). To 200 μl of the tissue homogenates, 800 μl of acidified (0.75 N HCl) isopropyl alcohol solution (1∶1 v/v) were added and were kept at 4°C for 24 h for DOX extraction. DOX was measured by the fluorescent emission of the solution (λ_ex_ = 480 nm, λ_em_ = 590 nm) in the cell lysate with Synergy HT Multi-Mode Micro plate Reader, BioTek, USA. The auto fluorescence's from the tissues were adjusted by subtracting the emission from the vehicle (PBS) treated tissues. For flow cytometry analysis, following mechanical disruption, tissue blocks were treated with 0.1 mg/ml Collagenase D (Roche) for 1 h at 37°C. RBC was lysed by RBC lysis buffer (8.3 g/l ammonium chloride in 0.01 M Tris-HCl buffer) and single cell suspension was prepared. Fluorescence histograms were then recorded with a BD FACS Calibur flow cytometer (Beckton Dickinson, U.S.A.) in FL3 channel. 20000 events were analyzed to generate each histogram.

### Tumor sample analysis

Formalin-fixed, paraffin-embedded primary tissues from liver, lung and spleen were obtained during therapeutic procedures from control, DL tumor bearing mice and the tumor bearing mice undergone indicated therapy. Hematoxylin and eosin-stained sections were examined for regions that contained tumor cells and stroma indicating the extent of metastasis. All studies were conducted using protocols approved by the Institutional Review Board of the University.

### Apoptosis Study in Organs after Therapy

Evaluation of apoptotic cell death in tumor, spleen, liver and lung cells by free doxorubicin or, by DOX-PCL_63_-b-PNVP_90_ micelles after therapy was assessed by binding FITC-conjugated Annexin V. In a single cell suspension of various organs, the percentages of FITC-conjugated Annexin V-positive cells were analyzed by BD FACS Calibur flow cytometer as described earlier (Beckton Dickinson, U.S.A.). For microscopic study, liver and spleen single cell suspension were washed with ice-cold PBS and stained with FITC-conjugated Annexin V and mounted on glass slides with a drop of mounting media to reduce fluorescence photo bleaching. The FITC-conjugated Annexin V positive cell and intracellular DOX localization were visualized under a fluorescence microscope (Nikon Eclipse 80i, Nikon, Japan).

### Statistical Analysis

Flow cytometry data were analyzed using Flow-Jo software (version 10.0.5; Treestar, Ashland, OR, USA). The mean ± SD were calculated for each experimental group (n = 3–5). Differences between groups were analyzed by unpaired Student's t-test and one- or two-way ANOVA analysis of variance depending on the requirement. One or two-way ANOVA followed by Holm-Sidak post-hoc multiple comparison tests was used to conduct pair wise comparisons using PRISM statistical analysis software (Graph Pad Software, Inc., San Diego, CA, USA). Significant differences among groups were calculated at P<0.05 or less (*P<0.05, **P<0.01, ***P<0.001, ****P<0.0001 in control versus experimental group). Statistical significance of differences in survival of the mice in different groups was determined by the log-rank test using Graph Pad PRISM software.

## Results and Discussion

The present study was motivated by the need to design a safe nano-carrier for the delivery of doxorubicin which could be tolerant to normal cells. PCL_63_-*b*-PNVP_90_ [13] was loaded with doxorubicin (6 mg/ml), and with 49.8% drug loading efficiency; it offers a unique platform providing selective immune responses against lymphoma. The delivery system proved to be a novel strategy, preventing metastasis in a highly aggressive lymphoma besides maintaining a minimum cardiotoxicity.

### Preparation and Characterization of Blank and DOX-PCL_63_-b-PNVP_90_ Miceller Nanoparticle

**Figure 1:**
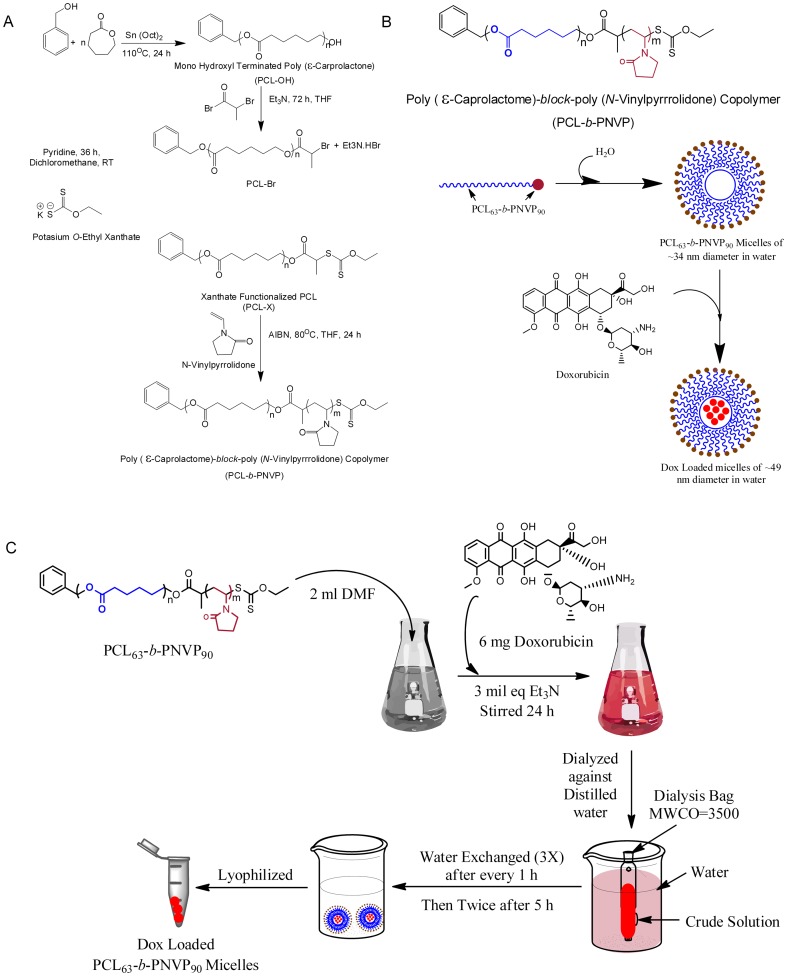
Synthesis and loading of Doxorubicin in PCL_63_-*b*-PNVP_90_. (A) Schematic illustration of the synthesis of well-defined amphiphilic poly (*ε*-caprolactone)-*b*-poly (*N*-vinylpyrrolidone) block copolymer via the combination of ROP and xanthate-mediated RAFT polymerization. (B) Schematic illustration of the formation of micelle or, drug-loaded micelle. (C) Details illustration of the synthesis of DOX- PCL_63_-*b*-PNVP_90_.

Well-defined amphiphilic block copolymer PCL_63_-*b*-PNVP_90_ containing hydrophobic PCL block made of 63 CL unit and hydrophilic PNVP block of 90 NVP unit [Mn (GPC) = 17,200 g mol^−1^, Mw/Mn = 1.39, critical micellar concentration in water as observed by fluorescence study using pyrene as probe  = 0.006 mg/ml] was successfully synthesized according to our recently published method (Figure 1A) [13]. Drug-free PCL_63_-*b*-PNVP_90_ micellar nanoparticle and DOX-PCL_63_-*b*-PNVP_90_ micellar nanoparticle were prepared by dialysis method as shown in Figure 1B and 1C. DOX is physically entrapped and stabilized in the hydrophobic PCL core of the micelles of PCL_63_-*b*-PNVP_90_ block copolymer via hydrophobic-hydrophobic interaction. The observed drug-loading content (DLC) and drug-loading efficiency (DLE) of DOX in the micelles of PCL_63_-*b*-PNVP_90_ are 19.0 and 49.8%, respectively, as quantified by UV-Vis spectroscopy at 451 nm (Figure S1 A-C). Loading of the drug in the micelle is evidenced by the increase in the size (TEM study) and hydrodynamic radius (Rh) (DLS study) of the drug-free micellar nanoparticle [from radius of 17 nm (TEM study) and hydrodynamic radius (Rh) of 42.5 nm (DLS study)] [13] towards the formation of the drug-loaded micellar nanoparticle [of radius of 24.5 nm (TEM study) and hydrodynamic radius (Rh) of 110 nm (DLS study)] (Figure S1 E-F). To study the effect of the pH of the release medium on the release kinetics of the drug from the drug-loaded micellar nanoparticle, we have chosen two phosphate buffer saline (PBS) solutions of pH 6.4 and 7.4. As shown in Figure S1 D, the observed DOX release (%) of drug loaded micelles at pH 7.4 and 6.4 are ∼19% and ∼37%, respectively. The drug release rate increases gradually up to initial 12 h and then becomes leveled off which may due to the strong interaction between drug and PCL hydrophobic block as well as due to the low solubility of the drug in the used PBS media. The observed rapid release of DOX loaded micelles at low pH is due to the presence of NH_2_ functional group in DOX. So, the observed accelerated drug release at low pH solution can be considered as an advantage for the antitumor drug delivery system.

### Growth Inhibition by DOX-PCL_63_-b-PNVP_90_ in Lymphoma Cells

**Figure 2:**
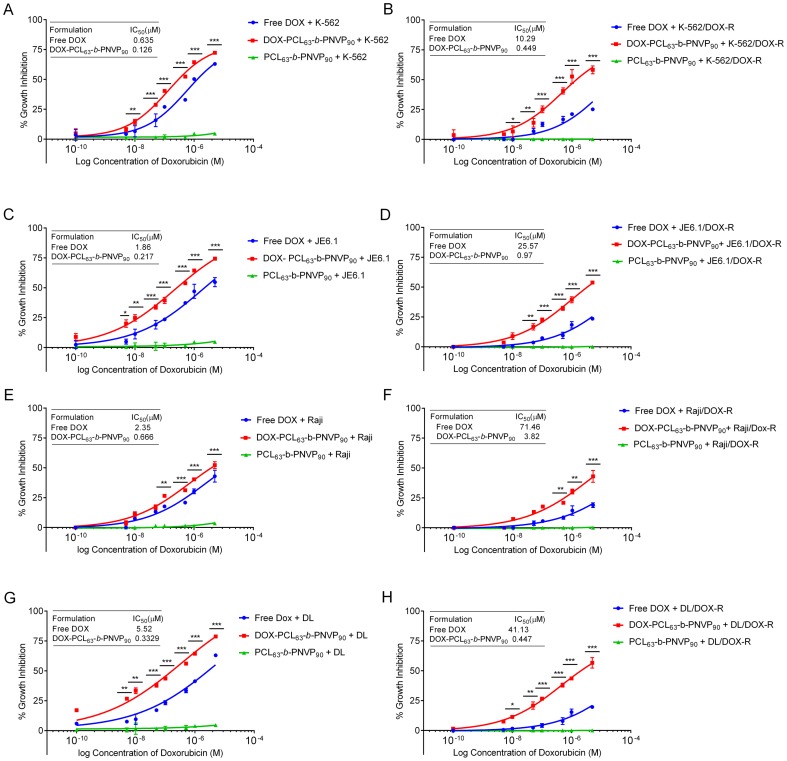
In vitro anti-tumor efficacy of DOX-PCL_63_-b-PNVP_90_. Graphs show doxorubicin concentration response on cell survival after treatment with free doxorubicin or, DOX-PCL_63_-b-PNVP_90_ in parental and doxorubicin resistant human erytholeukemic cell line K-562 (A and B), T cell leukemia line JE6.1 (C and D), Burkitt lymphoma cell line Raji (E and F) and mouse lymphoma cell line, Dalton lymphoma (DL) (G and H). Data presented as mean ± SD, n = 5. Differences in IC_50_ values between parental and doxorubicin resistant cell lines are mentioned. (*P<0.05, **P<0.01, ***P<0.001, ****P<0.0001 in control versus experimental group).

We performed 48 h growth inhibition study by the DOX-PCL_63_-*b*-PNVP_90_ against parental and DOX-resistant human (K-562, JE6.1, Raji) and mice lymphoma cells (DL) (Figure 2). Compared to free doxorubicin (DOX), DOX-PCL_63_-*b*-PNVP_90_ shows significantly higher growth inhibition in a concentration dependent manner against the cells tested. K-562 (6.62±2.67 vs. 14.39±1.23, p = 0.0227, n = 6), JE6.1 (11.22±4.08 vs. 24.77±2.69, p = 0.007, n = 6), and DL (9.52±4.27 vs. 33.36±2.32, p<0.0001, n = 6) responded better with significantly higher growth inhibition compared to free DOX (Figure 2 A, C, E, and G) above the doxorubicin concentration 0.01 μM. Raji appears to be little less sensitive although effect of DOX- PCL_63_-*b*-PNVP_90_ is higher in Raji compared to free DOX (20.64±0.625 vs. 31.25±0.194, p = 0.022, n = 6) at DOX concentration of 0.5 μM (Figure 2E). Carrier it-self has no effect on growth inhibition in any of the cell line tested. DOX-resistant variants of the above cell lines are refractory to free DOX (26.97±0.97 vs. 8.53±1.8, 23.51 vs. 7.27±6.69, 17.66±1.17 vs. 5.53±1.31 & 23.35±1.7 vs. 4.19±1.22, mean ± SD, n = 6 for resistance vs. sensitive K-562, JE6.1, Raji, DL cell lines respectively). On the other hand, the cell lines are sensitive to DOX-PCL_63_-*b*-PNVP_90_ (40.40±1.22 vs. 30.53±2.7, 39.39±2.512 vs. 28.43±1.64, 26.52±1.44 vs. 20.69±1.17& 43.62±0.627 vs. 35.7±1.22 mean±SD, n = 6 for resistance vs. sensitive K-562, JE6.1, Raji, DL cell lines respectively) at comparable molar concentrations of DOX (0.1 μM). IC_50_ for DOX-resistant K562, JE6.1, Raji and DL increases to 10.29±0.140, 25.57±0.440, 71.46±1.63 and 41.13±3.7 μM from 0.635±0.018, 1.86±0.061, 2.35±0.071 and 5.52±0.086 (mean ± SD, n = 4) μM respectively from their DOX sensitive counterpart (p = 0.0001) (Figure 2 B, D, F, and H). Free DOX unable to control the tumor cell growth at a concentration of 10^−6^ M, a concentration in which DOX-PCL_63_-*b*-PNVP_90_ shows remarkably high growth inhibition of all the resistant variants tested. Biodegradable micelles based on amphiphilic block copolymers such as poly (*ε*-caprolactone) (PCL) have emerged as one of the most promising nano system for controlled and site-specific delivery of potent lipophilic anticancer drugs like doxorubicin due to their proven biocompatibility and FDA approval for medical uses [18]–[21]. Besides reducing drug induced side effects, these micelles enhance water solubility, bioavailability and increase drug accumulation in tumors via enhanced permeability and retention (EPR) effect [22]. Stimuli (pH, temperature etc.) sensitive biodegradable polymers have recently been developed with faster intracellular drug release feature in tumor cells and ability to reverse multidrug resistance (MDR) in cancer cells [23], [24]. Our data shows that DOX-PCL_63_-*b*-PNVP_90_ works better in lower pH compared to higher pH suggesting its better suitability in acidic environment inside the tumor cells (Figure S1 D). This was supported by the susceptibility of human and murine lymphoma cells against DOX-PCL_63_-*b*-PNVP_90_ with respect to growth inhibition (Figure 2).

### Cellular Cytotoxicity by DOX-PCL_63_-b-PNVP_90_ in Lymphoma Cells

**Figure 3:**
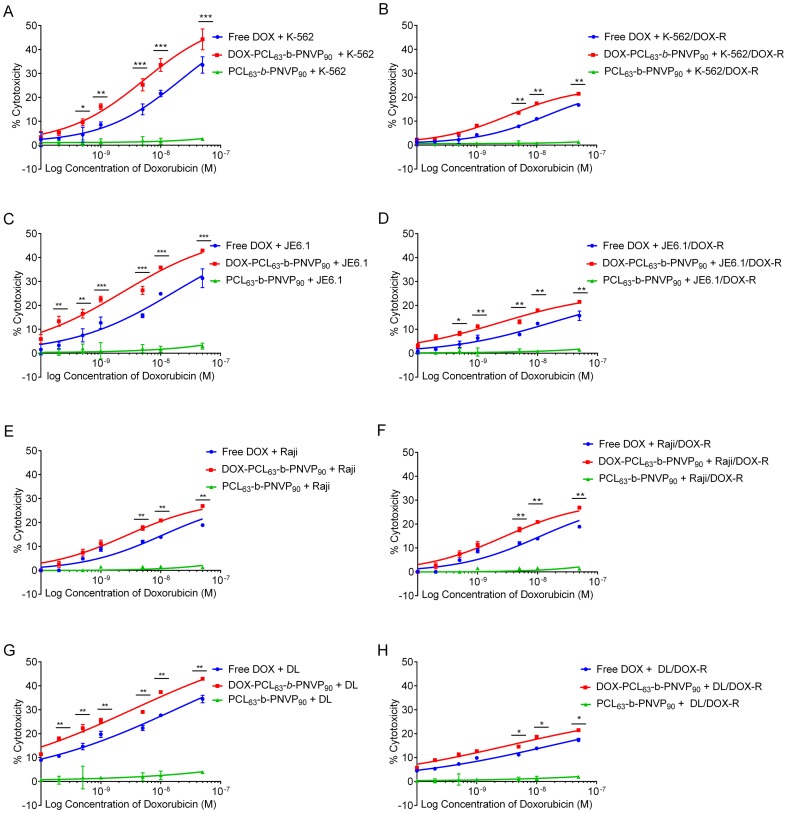
Cytotoxicity of DOX-PCL_63_-b-PNVP_90_. Cytotoxic effect of DOX-PCL_63_-b-PNVP_90_ against parental and DOX-resistant lymphoma cells was determined by 18 h LDH release assay. Cells were incubated with free polymer (carrier), DOX-PCL_63_-b-PNVP_90_ micelles or free DOX solution at different concentrations (0.0001, 0.0002, 0.0005, 0.001, 0.01, 0.05, 5 μM) for 18 h followed by measurement of the released LDH according to the manufacture's protocol. (A and B) K-562 and K-562/DOX-R, (C and D) JE6.1 and JE6.1/DOX-R (E and F) Raji and Raji/DOX-R (G and H) DL and DL/DOX-R. (*P<0.05, **P<0.01, ***P<0.001, ****P<0.0001 in control versus experimental group) data represents mean ± SD, n = 3.

The growth inhibition data suggested that DOX-PCL_63_-*b*-PNVP_90_ has significantly higher anti-tumor potential with respect to the reduction in the growth of lymphoma cells from human and mice. We wanted to know whether the growth inhibition is preceded by direct cytotoxicity of the tumor cells. Our results suggest that DOX-PCL_63_-*b*-PNVP_90_ is highly cytotoxic compared to free DOX at each molar concentrations of DOX tested (mean difference 5.182, 9.033, 2.409, 7.653 & p values 0.009, 0.0018, 0.0039, 0.006 respectively for K-562, JE6.1, Raji & DL at a DOX concentration of 0.001 μM (Figure 3A, C, E, G). The carrier it-self does not show cytotoxicity against any of the cell lines tested. Similar to the growth inhibition, resistant variants of all the cell lines are tolerant to free DOX but remain susceptible to DOX-PCL_63_-*b*-PNVP_90_ at all the concentrations tested (p = 0.03, 0.0129, 0.021, 0.0114 at concentration of 0.001 μM) (Figure 3B, D, F, and H). The development of multidrug resistance (MDR) has been a major impediment to the success of cancer chemotherapy due to its requirement for high dose regimens accompanied with increased toxicity and need for specific drug release rates during disease evolution [25]–[27]. Our formulation of DOX-PCL_63_-*b*-PNVP_90_ shows significant growth inhibition and cytotoxicity against resistant variants of human and mice lymphoma cells, suggesting broad spectrum usefulness of the compound (Figures 2 and 3).

### Cellular Uptake Studies

**Figure 4:**
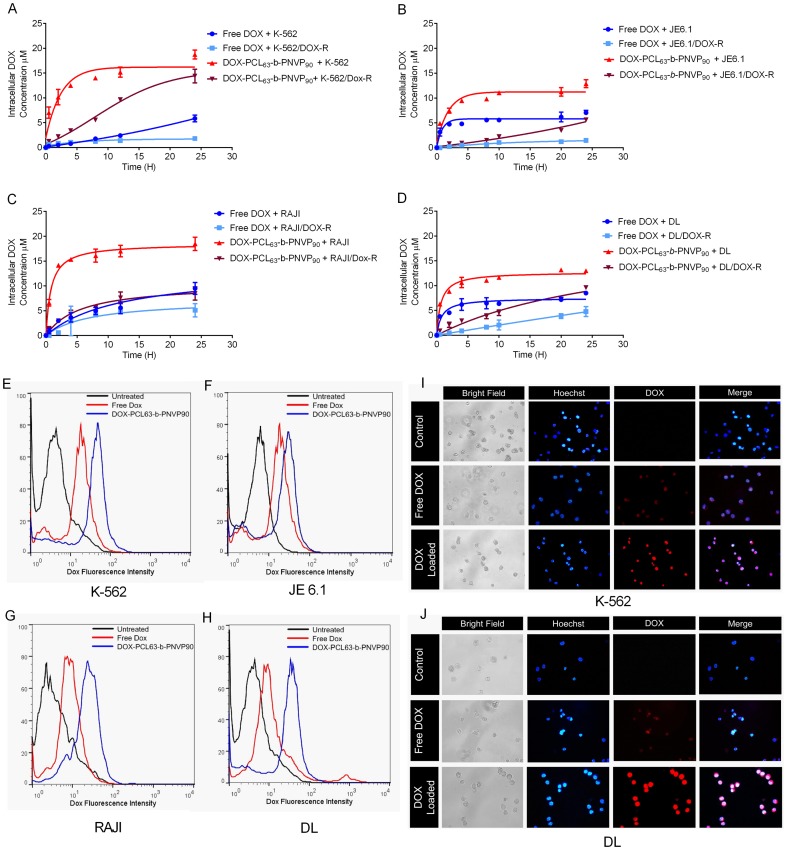
Time dependent uptake of free doxorubicin and DOX-PCL_63_-b-PNVP_90_ micelles. Uptake of free DOX or DOX-PCL_63_-b-PNVP_90_ micelles by parental and doxorubicin-resistant K-562 (A). JE6.1 (B) Raji (C) or, DL (D) cells were studied using fluorescence plate reader. Intracellular DOX concentration (μM) was measured in triplicate at different time points up to 30 h. Data presented as Mean ± SD, n = 4. Flow cytometric measurements of cellular DOX levels in parental K-562 (E) JE6.1 (F), Raji (G) and DL (H) (n = 3). Temporal uptake of free DOX or DOX-PCL_63_-b-PNVP_90_ micelles into K-562 (I) and DL (J) cells were studied by incubating in 24-well plates at a concentration of 50,000 cells per well. Representative images shown were obtained using a fluorescence microscope Eclipse 80i (Nikon, Japan) (Plan Fluor, 40X, NA 0.75 objective) equipped with blue and red filters for, Hoechst and Doxorubicin respectively (n = 3).

We generated DOX resistant K-562, JE6.1, Raji and DL by continuous culture of the tumor cells in medium containing IC_50_ doses of DOX and selected to test the efficiency of DOX-loaded PCL_63_-*b*-PNVP_90_ in overcoming the DOX resistance. DOX resistance was documented by the huge increase in the IC_50_ doses of each cell lines and their tolerance to the doses of DOX, usually causes death of the non-resistant variants (Figures 2 and 3). We next demonstrated whether DOX-PCL_63_-*b*-PNVP_90_ can increase the drug accumulation and retention in normal and resistant variants of the cell lines tested. Free DOX or DOX-PCL_63_-*b*-PNVP_90_ in culture medium was incubated with either resistant or parental variants of K-562, JE6.1, Raji or, DL at concentration of 2.0 μM of DOX for different time intervals from 0 to 30 h. The medium was discarded by washing in PBS and intracellular DOX accumulation was determined by analyses of DOX concentration in the cell lysates, which were normalized to total cellular protein content of the cells. It should be mentioned that the fluorescence intensity of DOX loaded PCL_63_-*b*-PNVP_90_ was similar to that of pure DOX at the same pH and molar concentration. Figure 4A–D illustrates the intracellular accumulation of doxorubicin (or equivalent) in parental and DOX resistant variants of K-562, JE6.1, Raji or, DL, respectively. Owing to the efflux of DOX, the intracellular concentration of DOX in parental K-562 cells was 5.85±0.665 μM per mg of protein after 24 h of incubation, which was significantly reduced (p = 0.260, n−3) in DOX-resistant K-562 (1.81±0.409 per mf of protein) cells after the incubation with free DOX for the same period of time. In contrast, a huge increase (14.07±0.335 μM per mg of protein) in intracellular accumulation of DOX was observed within 5 h in parental K-562 cells treated with DOX-PCL_63_-*b*-PNVP_90_ and remained stable for 25 h when data collection was stopped. For the DOX-resistant K-562, the intracellular DOX-level steadily increases as the time progresses and reached nearly the same level as in the parental K-562. Incorporation of free DOX in DOX-resistant K-562 was less compared to the parental K-562 and remained at the base line throughout the incubation period (1.027±0.069 at 4 h to 1.81±0.409 at 24 h). Similar results were observed with all the other cell lines mentioned above. We then qualitatively studied the intracellular DOX concentration in parental variants of the cell lines following incubation with free DOX or DOX-PCL_63_-*b*-PNVP_90_ by FACS. As observed in Figure 4 E–H, the DOX incorporation was significantly higher in all the cell lines tested confirming our quantitative determination of DOX incorporation. With DOX-resistant variants, we observed similar results as described by uptake studies (data not shown). We extended our observation of DOX-uptake at microscopic level for visualizing the presence of significantly higher DOX concentration intracellularly in K-562 and DL. As observed in Figure 4I and 4J, the intracellular level of DOX is significantly higher in cells treated with DOX-PCL_63_-*b*-PNVP_90_ compared to free DOX suggesting high drug delivery efficiency of the compound.

### Internalization and Intracellular Drug Release Behavior of DOX-PCL_63_-b-PNVP_90_

**Figure 5:**
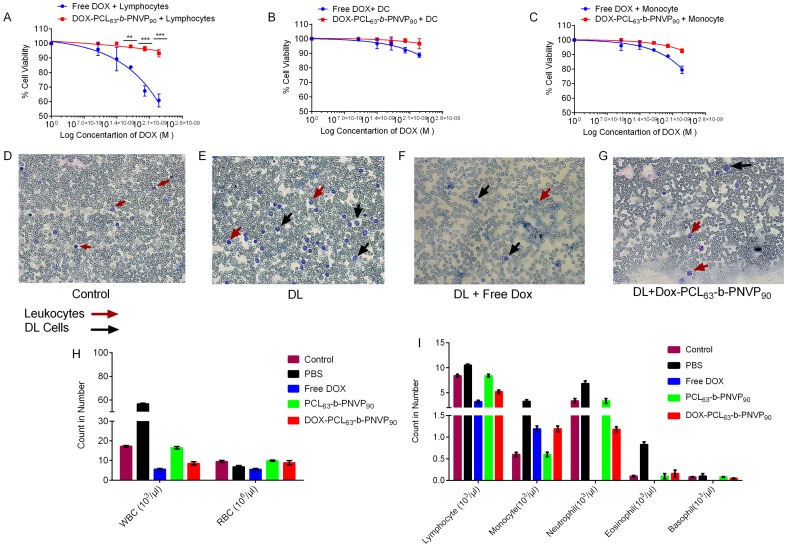
Effect of free DOX and DOX- PCL63-b-PNVP90 on normal Cell viability. Normal human lymphocytes (A), DC (B), and monocytes (C) were treated with serial concentrations of doxorubicin (0.0001, 0.005, 0.01, 0.05, 0.1, 0.5, 1, and 5 μM). Plates were incubated at 37°C, 5% CO_2_, for 48 h. The cell viability was measured by XTT assay kit (Cell Signaling, USA). Data shown as mean ± SD, n = 3. Effects on leukocytes number following in vivo administration of free DOX and DOX-PCL_63_-b-PNVP_90_ was studied in normal mice (D) tumor bearing mice (E) mice treated with free DOX (F), or DOX- PCL_63_-b-PNVP_90_ (G). RBC (H) and differential leukocyte count (I) of individual treatment are shown, mean ± SD, n = 3. (*P<0.05, **P<0.01, ***P<0.001, ****P<0.0001 in control versus experimental group).

We next demonstrated that DOX could be released from DOX-PCL_63_-*b*-PNVP_90_ in response to the intracellular acidic microenvironment during or after its accumulation in tumor cells. Endocytosis is known as one of the important entry mechanisms for various extracellular materials, particularly nanoparticles, which is energy dependent and can be hindered when incubation is performed at low temperatures (e.g., 4°C instead of 37°C) [28]. We found that free DOX demonstrate significantly higher release behavior compared to DOX-PCL_63_-*b*-PNVP_90_ (Figure S2). We also studied the DOX release pattern in normal cells like lymphocytes and purified dendritic cells (DC). Our results suggest that intracellular DOX concentration rises to its maximum levels (14.21±1.382 μM) between 5–10 h and drop very sharply to a level as low as 3.5±0.320 μM (Figure S3). Surprisingly lymphocytes retain free DOX compared to DOX-PCL_63_-*b*-PNVP_90_ for longer period of time. DC on the other hand does not retain either free DOX or DOX-PCL_63_-*b*-PNVP_90_ for long. DOX-PCL_63_-*b*-PNVP_90_ is significantly less toxic and exhibits nearly complete tolerance by the normal lymphocytes, dendritic cells and monocytes with very low intracellular retention (Figure 5). Free doxorubicin on the other hand shows extreme toxicity against lymphocytes and moderate toxicity against monocytes and dendritic cells (Figure 5). This observation is immensely significant with respect to immune response associated with the drug initiated tumoricidal effect where intact innate and adaptive immune repertoire is must for achieving the desired goals.

### Tolerance of Normal Cells to DOX-PCL_63_-*b*-PNVP_90_

Unlike tumor cells, white blood cells from normal human PBMC showed tolerance to DOX-PCL_63_-*b*-PNVP_90_ (Figure S4 A–C). Viability of cellular fractions like lymphocytes, dendritic cells (DC) and monocytes remain unaltered in the presence of DOX-PCL_63_-*b*-PNVP_90_. In contrast, free DOX found to be highly cytotoxic against lymphocytes (% viability of lymphocytes 60.87±4.44 vs. 93.26±2.538, p<0.0001 at concentration 1 μM) and also reduces viability of DC and monocytes as well at comparable molar concentrations of DOX tested (Figure 5 A–C). We also checked the fate of WBC and RBC upon treatment with free DOX or DOX-PCL_63_-*b*-PNVP_90_. Our result suggests that in DL tumor bearing mice, WBC number increases significantly (17.20±4.12×10^3^/μl to 57.60±6.13×10^3^/μl, control vs. DL) while RBC number decreases (9.5±0.590×10^6^/μl in control to 6.80±0.68×10^6^/μl in DL). In case of free DOX treatment, WBCs virtually vanishes (5.6±0.378×10^3^/μl) with dramatic reduction in RBC as well. In contrast, with DOX-PCL_63_-*b*-PNVP_90_ treatment, WBC number decreases slightly (8.50±1.06×10^3^/μl) while RBC numbers remains similar to normal mice (Figure 5 D–H). Differential counting of WBCs suggests a sharp decline in lymphocytes and neutrophils count upon treatment with free DOX while the cells are tolerant with respect to DOX-PCL_63_-*b*-PNVP_90_ treatment (Figure 5 I).

### Induction of Tumor Cell Apoptosis by DOX-PCL_63_-b-PNVP_90_

**Figure 6:**
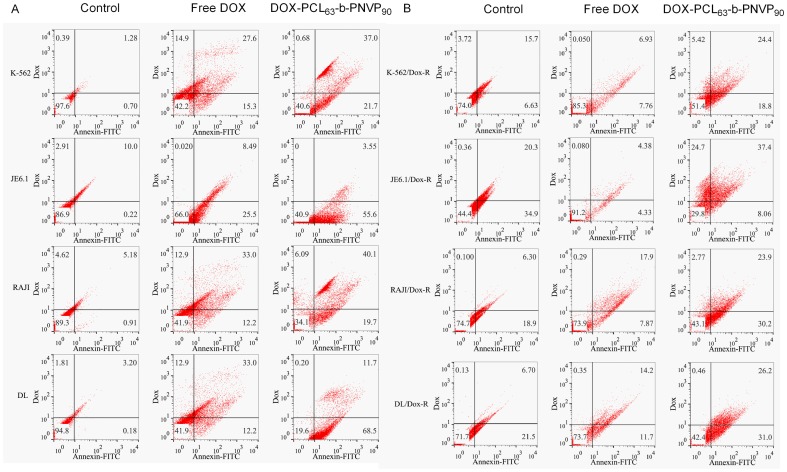
Induction of apoptosis by DOX-PCL_63_-b-PNVP_90_ micelles. The effect of free DOX or, DOX-PCL_63_-b-PNVP_90_ on the induction of apoptosis in parental (A) and doxorubicin resistant (DOX-R) (B) tumor cells was quantified by flow cytometric analysis of Annexin V FITC staining in tumor cells following 18 h treatment with indicated drugs. The lower left (LL) quadrant represents live/healthy cells, the lower right (LR) quadrant represents early apoptosis, and the upper right (UR) represents cells in late apoptosis, while the upper left (UL) represents the percent DOX uptake. The percentage of cells undergoing early or, late apoptosis is indicated in the respective quadrate. Data shown are mean ± SD, n = 3.

Broad spectrum growth inhibition by DOX-PCL_63_-*b*-PNVP_90_ raises the question whether it also causes apoptosis of tumor cells and if so whether it induces cell death in DOX-resistant tumor cells, which become refractory to any concentrations of DOX. Apoptosis was determined by monitoring changes in cell size and externalization of phosphatidylserine by flow cytometry after exposure to FITC-labeled Annexin V according to the manufacturer's instructions. The cells were harvested, stained with FITC-labeled Annexin V and analyzed by flow cytometry using the Cell Quest software program. A minimum of 10,000 cells was analyzed in each case with triplicate determinations. Our results shows that tumor cells behave differently with respect to annexin positivity when treated with DOX-PCL_63_-*b*-PNVP_90_, exhibiting significantly higher levels of apoptosis compared to DOX alone (Figure 6A). Annexin positivity in K-562 cells treated with DOX-PCL_63_-*b*-PNVP_90_ is higher (6.4% increase) compared to DOX alone. Annexin positivity in DL cells increases from 12% in free DOX treatment to 68%, when DL cells were treated with DOX-PCL_63_-*b*-PNVP_90_. Similar results were observed in all the other cell lines tested (Figure 6A). DOX-resistant cells are tolerant to DOX however; remain susceptible to DOX-PCL_63_-*b*-PNVP_90_ mediated apoptosis although percent Annexin positivity is significantly lower compared to the parental variants (Figure 6B). Percent Annexin positivity in K-562/DOX-R is 19% in cells treated with DOX-PCL_63_-*b*-PNVP_90_ compared to 8% treated with DOX alone. Huge difference was also observed in Annexin V-DOX double positive cells (7% in DOX treatment compared to 24% in DOX-PCL_63_-*b*-PNVP_90_ treatment) (Figure 6B). Similar results were obtained with other resistant cell lines including DL/DOX-R (Figure 6B).

We also qualitatively assessed the apoptosis in parental and DOX resistant K-562 and DL to document the events of apoptosis. There is an abundance of Annexin V positive cells in parental K-562 treated with DOX-PCL_63_-*b*-PNVP_90_ which is significantly higher compared to free DOX treatment. Resistant variants of the K-562 exhibits tolerance to free DOX but become susceptible to DOX-PCL_63_-*b*-PNVP_90_ (Figure S4). Similar results were obtained in DL cells under identical treatment (Figure S4).

### Clonogenic Survival Assay

**Figure 7:**
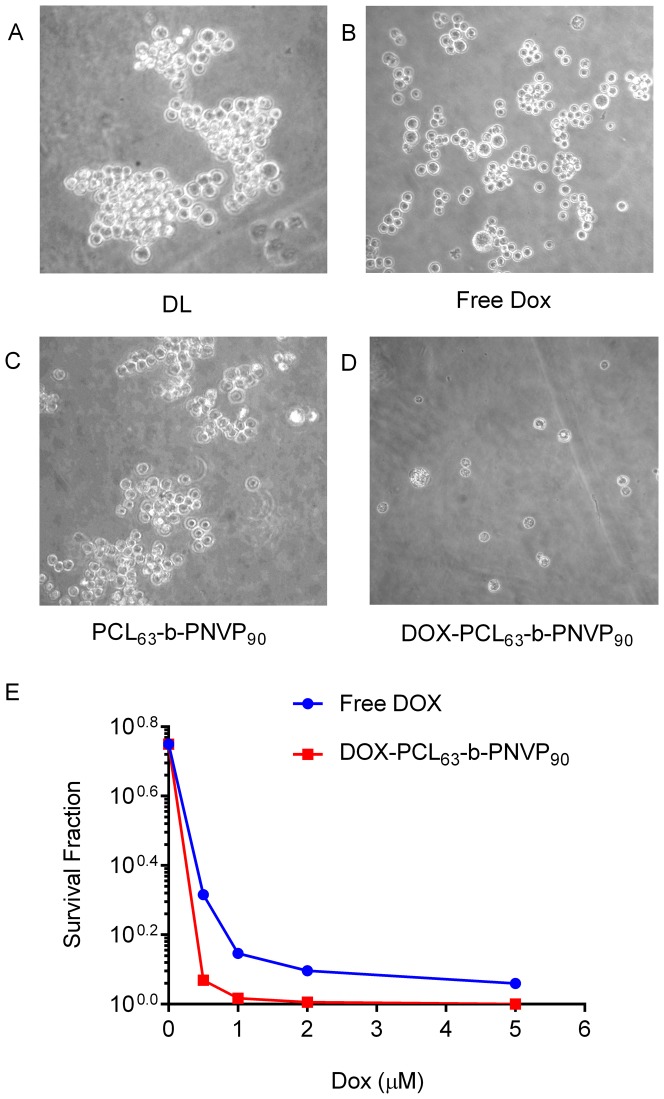
DOX-PCL_63_-b-PNVP_90_ hinders colony formation in DL. Clonogenic assay was performed in six-well plates following seeding of 100 cells with clones produced by DL tumor cells. Untreated controls and PCL_63_-b-PNVP_90_ treated cells formed colonies (A and C). Smaller size colonies with less number of cells were observed following doxorubicin (0.5 μM) treatment (B). Cells treated with DOX-PCL_63_-b-PNVP_90_ treatment (0.5 μM) unable to form colony (D). Survival analysis of DL cells treated with free DOX (Red Line) or, DOX-PCL_63_-b-PNVP_90_ (Blue line), (p = 0.01) (E). DOX-PCL_63_-b-PNVP_90_ induced growth inhibition in DL cells is caspase dependent. Growth inhibition study in presence and absence of pan caspase inhibitor ZVAD-FMK for 48 h (n = 3, mean ±SD). MTT assay was performed as described above (G).Data presented as mean ± SD, n = 3.

In addition to the rapid Annexin V assay, a clonogenic survival assay was also employed to assure that we were detecting a true commitment to die and not just early, potentially reversible, changes in the cells treated with either DOX or DOX-PCL_63_-*b*-PNVP_90_. Our results suggest that with DOX-PCL_63_-*b*-PNVP_90_ treatment, the number of colonies of DL cells become negligible compared to free DOX (Figure 7A–D). Analysis of survival fraction suggest that with DOX-PCL_63_-*b*-PNVP_90_ treatment, there is a sharp decline compared to free DOX at a concentration of 1–2 μM of DOX (Figure 7E).

### In Vivo Antitumor Activity

**Figure 8:**
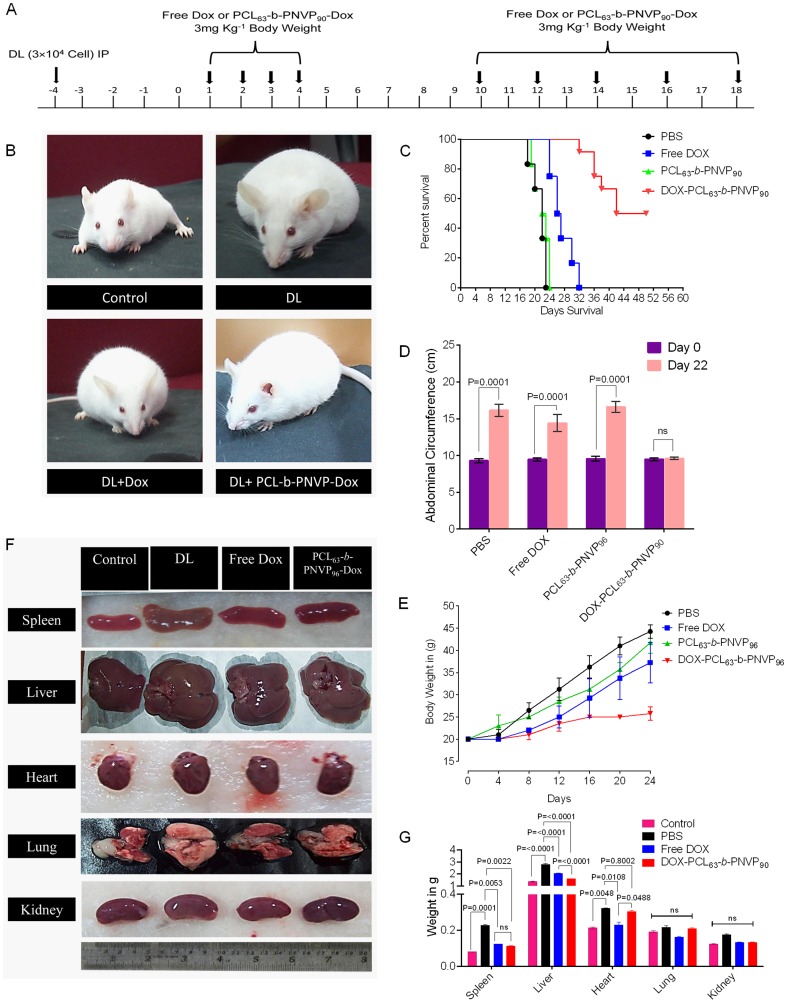
In vivo anti-tumor role of DOX-PCL_63_-b-PNVP_90_ micelles. Therapy started at 96×10^4^ tumor cells (DL) were injected i.p. in to AKR mice (n = 12/group) and referred as day 0. Lymphoma bearing animals were injected with nine doses of free DOX or, DOX-PCL_63_-b-PNVP_90_ micelles at a dose of 3 mg kg^-1^ of body weight doxorubicin as indicated by the arrows for 3 weeks (A). All together 9 doses were given in PBS; out of 9 doses, 4 doses were given every day (from day 0 to Day 4) and remaining 5 dose were given from day 10 at an interval of 48 h and continued up to day 18. The animals in study responded to the therapy with DOX-PCL_63_-b-PNVP_90_ (B). Kaplan-Mayer survival analysis of tumor bearing mice was performed following therapy and was analyzed for the percent survival up to day 50 post tumor transplant by log-rank test using Graph Pad PRISM software (C). Abdominal circumference (D) and body weight (E) are depicted demonstrating the effects of the treatment on the tumor growth of the animals. Representative images of spleen, liver, heart, lung, and kidney from each treatment group (F) and the corresponding weight of excised organ following drug treatment compared to untreated control are shown (G) n = 3.

**Figure 9:**
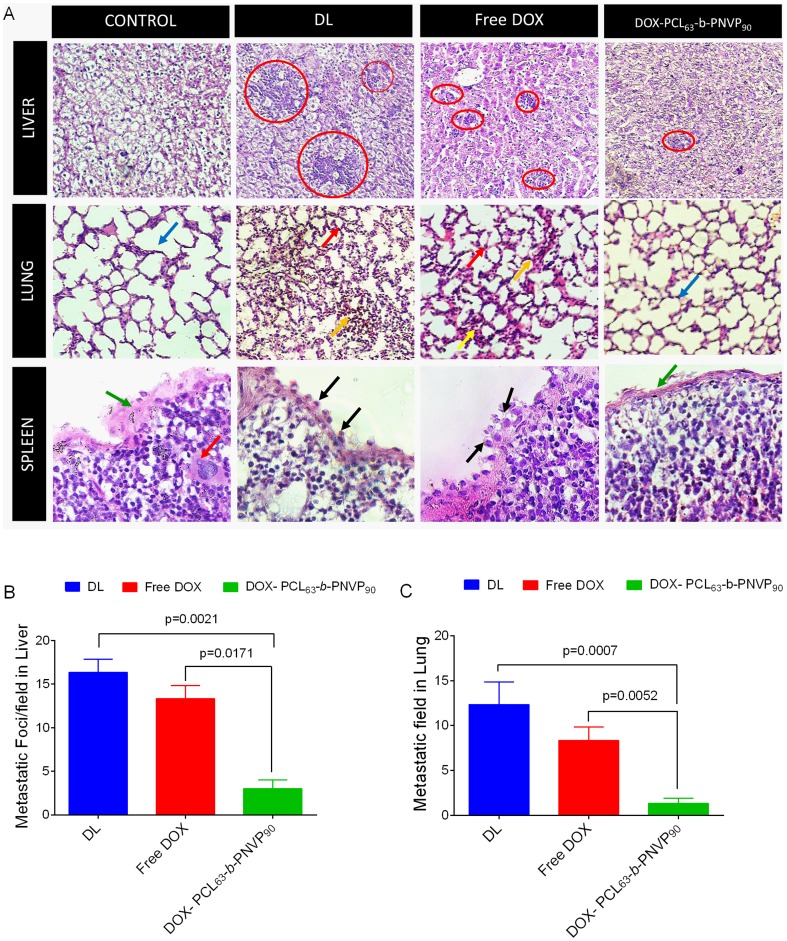
Histo-pathological analysis of tumor in vascularized organs. Images of haematoxylin and eosin (H&E) staining of Liver, Lung & Spleen tissues from normal (Control), tumor bearing (DL) or treated groups (Free DOX or DOX+PCL_63_-*b*-PNVP_90_) are shown. In liver infiltrated metastatic lymphoid cell (Red circle) are shown. Loss of normal architecture & recruitment of neutrophils (Red Arrow) & Evidence of haemorrhage and necrosis (yellow arrow) in lungs of treated group compared to normal (blue arrows). Normal spleen is with intact capsule (green arrow), no invasion by malignant cell, with hematopoietic cell (red arrow). In DL, sub-capsular sinus is infiltrated with malignant cell (black arrow) and in treated group, capsule invasion by malignant cells is observed. (A).Tumor metastatic foci/field (arrows) were counted (B & C), mean± SD, n = 4 are shown.

Therapeutic efficacy of DOX-PCL_63_-*b*-PNVP_90_ was validated in DL tumor model. Randomly sorted mice (female, 4-6 weeks old) were transplanted with tumor intraperitoneally and were sorted in to 4 groups each with 12 mice and treated with 4 daily injections of (i) phosphate buffer saline (PBS) (ii) free doxorubicin (3 mg/kg of body weight), (iii) vehicle control or (iv) DOX-PCL_63_-*b*-PNVP_90_ (equivalent to 3 mg/kg of doxorubicin dose) after 96 hours post tumor challenge (marked as day 0). A second set of 5 injections were given to the animals from day 10 to day 18 with a gap of 48 h (Figure 8A). The mice injected with PBS or, vehicle control formed large intraperitoneal ascitis by day 12 post tumor transplant, which continued with increasing size as the day progresses. Mice treated with free DOX also develops tumor and forms similar ascitis at day 20. In contrast, mice treated with DOX-PCL_63_-*b*-PNVP_90_ develops tumor at a significantly slower rate and develops tumor at day 30. All the mice in PBS and vehicle control groups died between day 22 and 24 from tumor progression. Mice in free doxorubicin group did not survive beyond the day 32. In contrast, 4 of 12 mice in the DOX-PCL_63_-*b*-PNVP_90_ group were alive at day 50 when the experiment was terminated. The animals in all the groups were sacrificed at the same time point to evaluate the effect of the treatments on tumor pathology. As shown in Figure 8B, treatment with vehicle control (DL) or, free doxorubicin (DL+DOX) forms large ascitis at day 18 compared to DOX-PCL_63_-*b*-PNVP_90_ (DL+DOX-PCL_63_-*b*-PNVP_90_). This was reflected in Kaplan-Mayer survival analysis showing prolonged survival of DOX-PCL_63_-*b*-PNVP_90_ treated mice compared to free DOX treatment (Figure 8C). This was coupled with a distinct reduction in abdominal circumference (16.13±0.814 in DL vs. 9.60±0.721 cm in DOX-PCL_63_-*b*-PNVP_90_ treated at day 22, p = 0.0001) and body weight in DOX-PCL_63_-*b*-PNVP_90_ group (25.7±1.5 gm./mice) compared to vehicle control (average 38 gm./mice) or, DOX treated mice (average 35.75±2.49 gm./mice) (Figure 8D & E). We also determined the weight of important vascularised organs in order to assess the effect of treatment on metastasis. Our results suggests that in liver, where DL tends to get metastasize shows remarkable therapeutic efficacy by reducing the organ weight, ostensibly by therapy with DOX-PCL_63_-*b*-PNVP_90_ (p<0.0001 between DOX and DOX-PCL_63_-*b*-PNVP_90_) (Figure 8F & 8G). Validation of therapeutic efficacy of DOX-PCL_63_-*b*-PNVP_90_ system in DL tumor model resulted in dramatic growth inhibition and prevention of metastasis, indicating the increase in therapeutic index of the formulation. High solubility of DOX-PCL_63_-*b*-PNVP_90_ and effect of tumor derived carboxylesterases could be responsible for release of doxorubicin and reduces its systemic toxicity [29]. Histopathological analysis of liver, lung and spleen clearly indicates the efficacy of the formulation in restricting the metastasis of the organs (Figure 9A). In liver, infiltrated metastatic lymphoid cell (red circle) are clearly visible in DL mice which are significantly reduced in mice treated with DOX-PCL_63_-*b*-PNVP_90_. Similarly, in lung normal architecture is lost due to tumor metastasis which is accompanied by the recruitment of neutrophils (red arrow) and evidence of haemorrhage and necrosis (yellow arrow). Upon treatment with DOX-PCL_63_-*b*-PNVP_90_, mice recovered compared to untreated group (blue arrows). In normal spleen, the capsule is intact (green arrow) throughout the periphery which is disintegrated in tumor bearing mice and not restored in mice treated with DOX only. In DL mice, sub-capsular sinus is infiltrated with malignant cell (black arrow) which is also visible in DOX treated group with capsule invasion by malignant cells. DOX-PCL_63_-*b*-PNVP_90_ treated mice restored the capsular architecture to a large extent (Figure 9A). Counting of metastatic foci and metastatic field in liver and lung sections clearly suggest significant reduction in metastasis following therapy with DOX-PCL_63_-*b*-PNVP_90_ (p<0.017 and 0.0052 between DOX and DOX-PCL_63_-*b*-PNVP_90_ treatment in liver and lung respectively) (Figure 9B & C).

### Enhanced uptake of DOX-PCL_63_-b-PNVP_90_ by Organs in Tumor Bearing Mice

**Figure 10:**
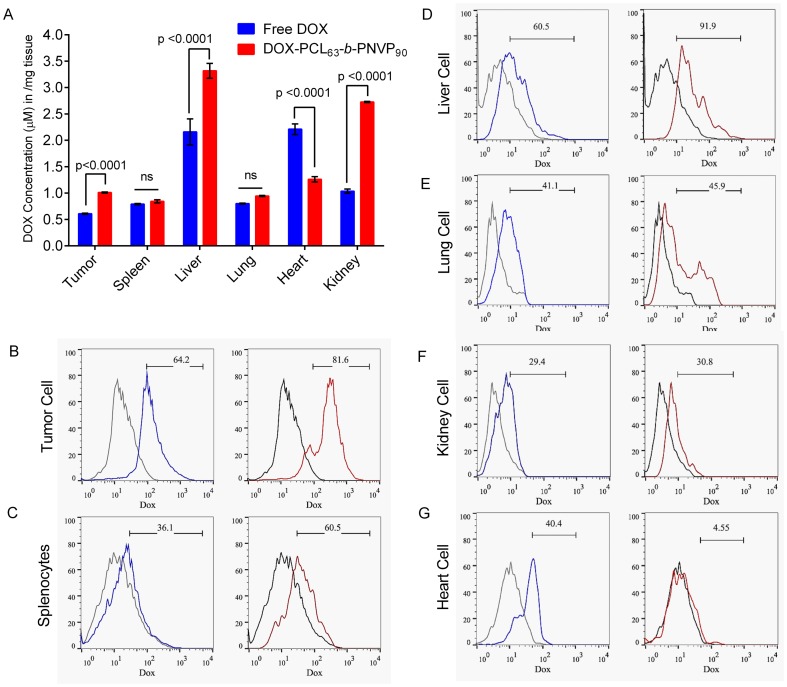
Doxorubicin distribution in organs following therapy with DOX-PCL_63_-b-PNVP_90_. Uptake of free DOX and DOX-PCL_63_-b-PNVP_90_ micelles by tumor cells and organs were studied using fluorescence plate reader (A). Data presented as mean ± SD of triplicate determination, n = 3. Distribution profile of DOX in Tumor cell (B), Spleen (C), Liver (D), Lung (E), Kidney (F), and Heart (G) by FACS analysis, n = 3.

Fluorescence spectroscopic analysis of doxorubicin level in heart showed very high accumulation in mice treated with free DOX compared to DOX-PCL_63_-*b*-PNVP_90_ despite no difference in organ weight. Unlike others [30], splenic accumulation of doxorubicin was not significant (0.790±0.012 vs. 0.844±0.029, p = 0.4667, n = 3) and no difference was observed in weight (data not shown) of the spleen between free DOX and DOX-PCL_63_-*b*-PNVP_90_ treated groups (Figure 10). This could be due to low concentration of doxorubicin used in our study. Additionally, kidney tissue showed significantly higher accumulation (1.037±0.638 vs. 2.729±0.011, p<0.0001, n = 3) of DOX-PCL_63_-*b*-PNVP_90,_ suggesting easy clearance of the formulation following metabolism. Liver and tumor cells however, showed very high accumulation. Therapy with DOX-PCL_63_-*b*-PNVP_90_ causes change in the accumulation of DOX in the targeted organs. The accumulation of DOX-PCL_63_-*b*-PNVP_90_ was enhanced in liver, kidney (p<0.0001) and lung (p = 0.0012) while in heart, DOX concentration is higher (p<0.0001) compared to DOX-PCL_63_-*b*-PNVP_90_ (Figure 10A). FACS analysis data supported the observation with major shift in accumulation of DOX in DOX-PCL_63_-*b*-PNVP_90_ treated mice which was diametrically different in case of heart (Figure 10G). These data suggest that DOX-PCL_63_-*b*-PNVP_90_ is nontoxic and perhaps causes very less damage to the heart tissue. We also analyzed the apoptosis in single cell suspension derived from spleen, liver and lung tissues following staining with Annexin V of the total cells. Microscopic analysis of total liver cells shows greater Annexin positivity derived from mice treated with DOX-PCL_63_-*b*-PNVP_90_ compared to free DOX (Figure S5 A). FACS analysis of cells derived from liver, spleen and lung showed enhanced apoptosis of cells in the target organs by DOX-PCL_63_-*b*-PNVP_90_ compared to DOX treatment alone (Figure S5 B).

Apoptosis observed in liver and spleen cells presented in Figure S5C are the mainly in tumor cells present in liver and spleen. No observable apoptosis was observed in control group. In case of DOX loaded micelle group, much more apoptosis is observed in tumor bearing mice. This is further supported by Figure S6, where we observe that free DOX causes cell death in normal mice spleen, liver and lung, significantly higher compared to DOX loaded polymer (normal mice were treated with free DOX or DOX loaded polymer). This indicates the general toxicity of free DOX while DOX- PCL_63_-*b*-PNVP_90_ remain significantly less toxic.

## Conclusion

In this study, we have demonstrated the potential application of doxorubicin-loaded poly (*ε*-caprolactone)-*b*-poly-(*N*-vinylpyrrolidone) micellar nano-carrier in cancer chemotherapy. Several components of this platform make it an attractive approach for facilitating future therapy in humans. Hydrophilic exterior and hydrophobic interior of our formulation offers entrapment of higher concentrations of doxorubicin to form micelles and get easy access through the cells leading to high efficiency of cellular uptake by endocytosis and subsequent acid responsive release in tumor cells. Assay with normal cells indicates reduced toxicity to WBCs and vital organs like heart while maintaining higher therapeutic efficacy, compared to commercial doxorubicin hydrochloride. DOX-PCL_63_-*b*-PNVP_90_, compared to free DOX, demonstrated antitumor potential against doxorubicin-resistant lymphoma cells indicating its possible application in drug resistant scenarios. Finally, DOX-PCL_63_-*b*-PNVP_90_ significantly inhibits in-vivo tumor growth in mice model of lymphoma through induction of apoptosis and prevention of metastasis. Designing of DOX-PCL_63_-*b*-PNVP_90_ opens a new way for treating cancer with higher efficacy and decreased side effects.

## Supporting Information

Figure S1 **Characterization of Doxorubicin loaded PCL_63_-** ***b*** **-PNVP_90_.** (A) UV-Vis absorbance spectra of DOX in DMF at different concentrations. (B) Corresponding calibration curve to determine the DOX concentration. (C) UV-Vis absorbance spectrum of DOX-loaded PCL_63_-b-PNVP_90_ micelles in DMF. (D) In vitro release profiles of DOX from DOX-loaded micelles in PBS of 6.4 and 7.4 pH. (E) Hydrodynamic radius and size of the PCL_63_-b-PNVP_90_ micelles as observed from DLS and TEM studies [13]. (F) Hydrodynamic radius and size of the DOX-loaded PCL_63_-b-PNVP_90_ micelles as observed from DLS and TEM studies.
(TIF)

Figure S2 **Intracellular DOX release and efflux study.** For the time course study, parental K-562 (A), JE6.1 (B), Raji (C) or, DL (D) cells were incubated in triplicate with 5.0 μM DOX-PCL_63_-b-PNVP_90_ micelles or free DOX for 12 h. To examine the efflux processes, the culture medium containing 5.0 μM DOX-PCL_63_-b-PNVP_90_ micelles was replaced by doxorubicin free medium after 12 h incubation. Cells were harvested after 2, 4, 8, 16 and 24 h following incubation. The fluorescence of DOX-PCL_63_-b-PNVP_90_ micelles in cells was measured using fluorescence plate reader. Data presented as mean ± SD, n = 4.
(TIF)

Figure S3 **Doxorubicin Uptake by normal cells.** For the time course study, dendritic cells (DC) (A) or lymphocytes (B) were incubated in triplicate either with 5.0 μM DOX-PCL_63_-b-PNVP_90_ micelles or free DOX for 12 h. To examine the efflux processes, the culture medium containing 5.0 μM free DOX or DOX- PCL_63_-b-PNVP_90_ micelles was replaced by doxorubicin free medium after completion of incubation. Cells were harvested after 2, 4, 8, 16 and 24 h following the incubation in DOX free medium. The fluorescence of DOX- PCL_63_-b-PNVP_90_ micelles in cells was measured using fluorescence plate reader. Data presented as mean ± SD, n = 3.
(TIF)

Figure S4 **Microscopic analysis of induction of apoptosis.** Temporal distribution of doxorubicin uptake in parental K-562 (A) DOX-R/K-562 (B) and parental DL (C), DOX-R/DL (D). Cells were treated with free Doxorubicin or, the DOX-PCL_63_-b-PNVP_90_ micelles with equivalent Doxorubicin at a concentration of 5.0 μM, in complete RPMI 1640 medium for 4 h at 37°C. The FITC-conjugated Annexin V positive cell and intracellular DOX localization was visualized under a fluorescence microscope (Nikon Eclipse 80i, Nikon, Japan). Representative images shown were obtained using a fluorescence microscope Eclipse 80i (Nikon, Japan) (Plan Fluor, 40X, NA 0.75 objective) equipped with green and red filters for FITC and DOX, respectively.
(TIF)

Figure S5 **Apoptosis of tumor cells in targeted organs upon trea^tm^ent with DOX- PCL_63_-b-PNVP_90_.** DOX-PCL_63_-b-PNVP_90_ micelle induces apoptosis in tumor cells metastasize in spleen (A) and liver (B) as judged by microscopic analysis. FACS analysis of Annexin V positive cells with doxorubicin uptake in tumor cell alone and tumor cells metastasize in spleen, liver and lung of mice treated with either free DOX or, PCL_63_-b-PNVP_90_ micelles (C), n = 3.
(TIF)

Figure S6 **Apoptosis of normal liver, spleen & lung cells upon treatment with DOX or DOX-PCL_63_-b-PNVP_90_.** Spleen, liver & lung cell apoptosis was assessed by FACS analysis of Annexin V positive cells following doxorubicin uptake. Normal mice (4 per group) were treated with either free DOX or, DOX-PCL_63_-b-PNVP_90_ micelles and were kept for 22 days. The mice were sacrificed after 22 days and spleen and liver cells were harvested. The cells were stained with Annexin V-FITC and analysed in FACS calibur as performed above. At least 10,000 cells were counted with triplicate determination.
(TIF)
