# Supplementary figures and images for: Targeted Delivery of Doxorubicin-Loaded Poly (ε-caprolactone)-b-Poly (N-vinylpyrrolidone) Micelles Enhances Antitumor Effect in Lymphoma
Source: PLoS One. 2014 Apr 8;9(4):e94309. doi: 10.1371/journal.pone.0094309 (PMC3979807; doi:10.1371/journal.pone.0094309)

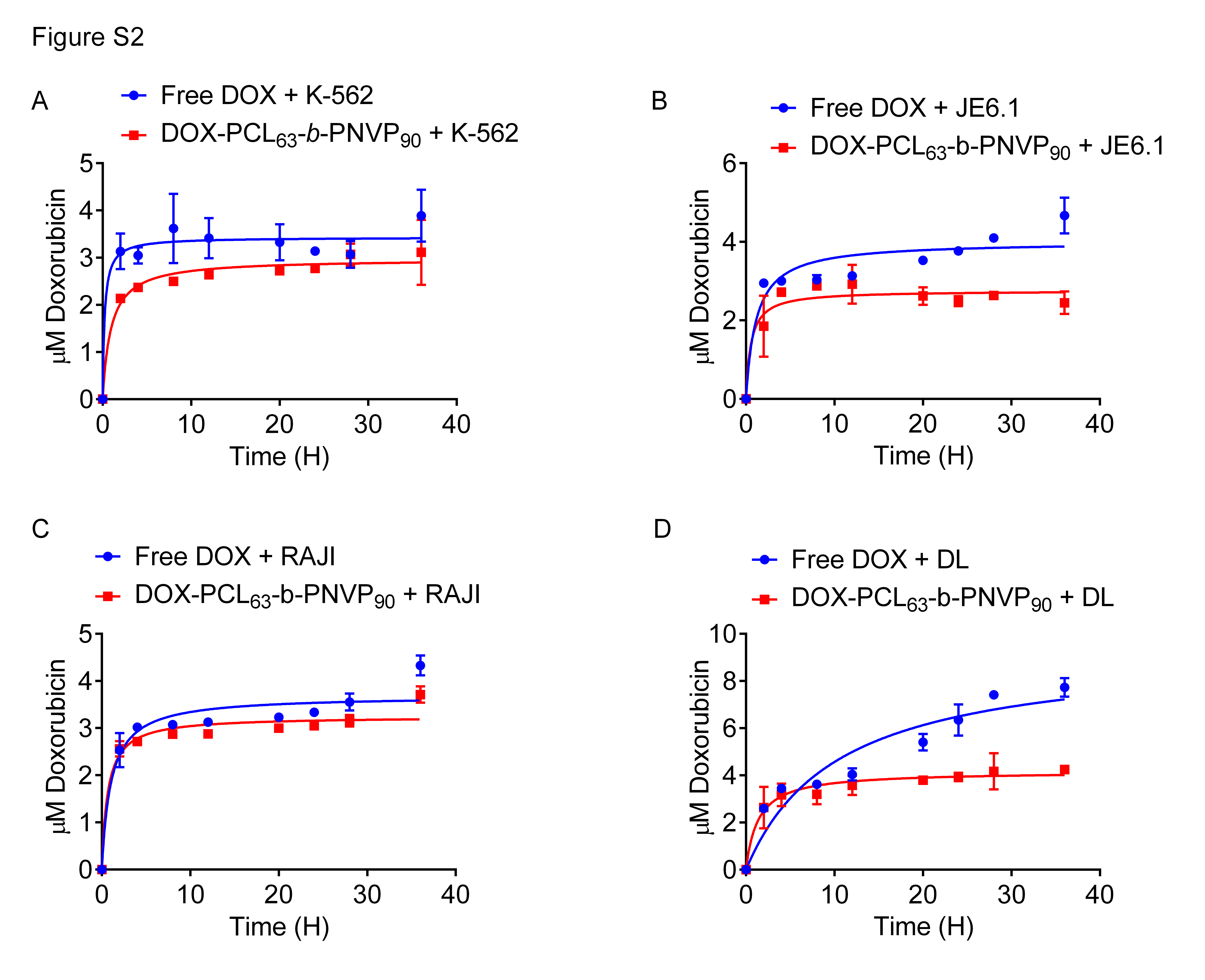

Supplement: Figure S2 — Intracellular DOX release and efflux study. For the time course study, parental K-562 (A), JE6.1 (B), Raji (C) or, DL (D) cells were incubated in triplicate with 5.0 μM DOX-PCL63-b-PNVP90 micelles or free DOX for 12 h. To examine the efflux processes, the culture medium containing 5.0 μM DOX-PCL63-b-PNVP90 micelles was replaced by doxorubicin free medium after 12 h incubation. Cells were harvested after 2, 4, 8, 16 and 24 h following incubation. The fluorescence of DOX-PCL63-b-PNVP90 micelles in cells was measured using fluorescence plate reader. Data presented as mean ± SD, n = 4. (TIF) [file pone.0094309.s002.tif]

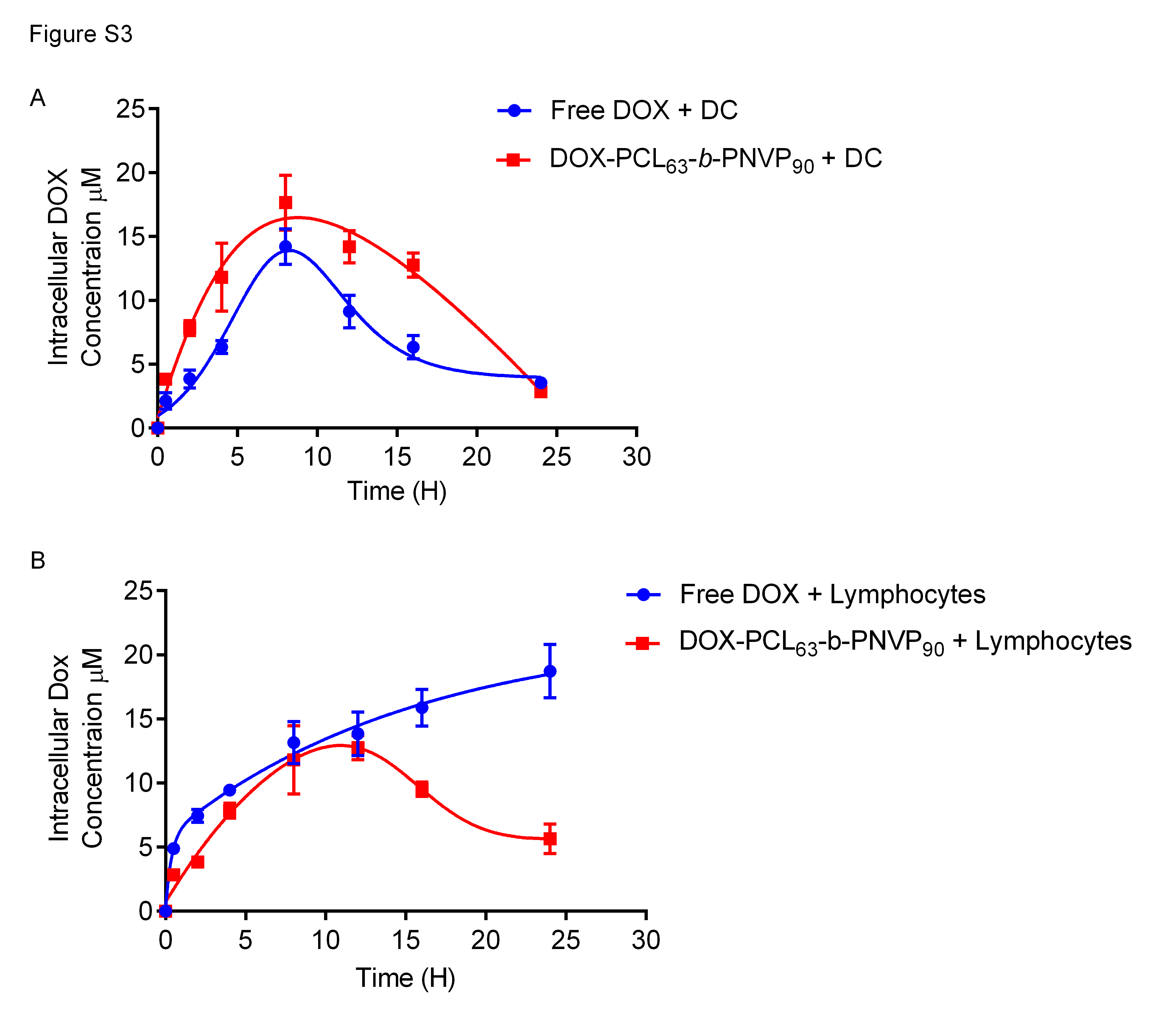

Supplement: Figure S3 — Doxorubicin Uptake by normal cells. For the time course study, dendritic cells (DC) (A) or lymphocytes (B) were incubated in triplicate either with 5.0 μM DOX-PCL63-b-PNVP90 micelles or free DOX for 12 h. To examine the efflux processes, the culture medium containing 5.0 μM free DOX or DOX- PCL63-b-PNVP90 micelles was replaced by doxorubicin free medium after completion of incubation. Cells were harvested after 2, 4, 8, 16 and 24 h following the incubation in DOX free medium. The fluorescence of DOX- PCL63-b-PNVP90 micelles in cells was measured using fluorescence plate reader. Data presented as mean ± SD, n = 3. (TIF) [file pone.0094309.s003.tif]

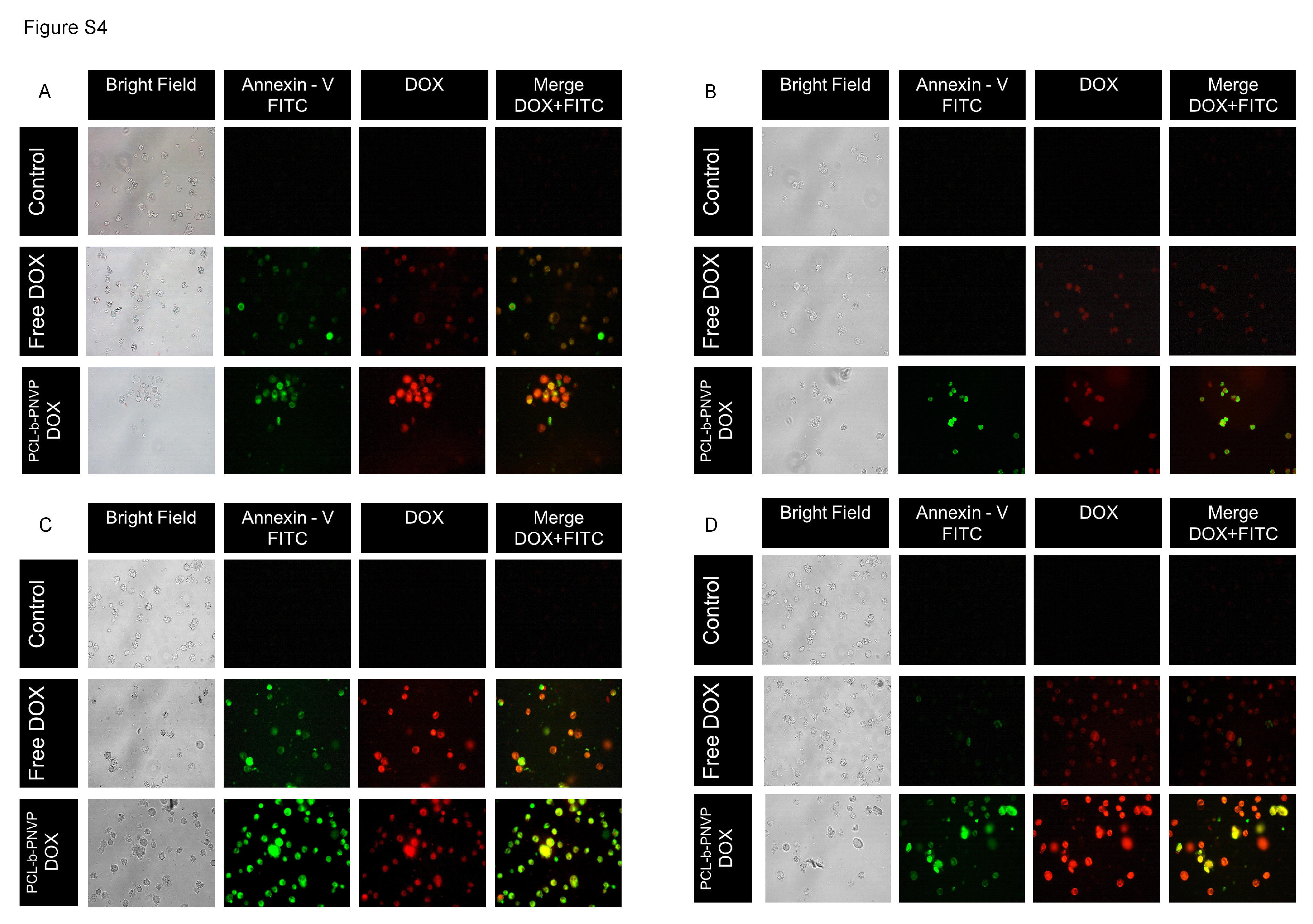

Supplement: Figure S4 — Microscopic analysis of induction of apoptosis. Temporal distribution of doxorubicin uptake in parental K-562 (A) DOX-R/K-562 (B) and parental DL (C), DOX-R/DL (D). Cells were treated with free Doxorubicin or, the DOX-PCL63-b-PNVP90 micelles with equivalent Doxorubicin at a concentration of 5.0 μM, in complete RPMI 1640 medium for 4 h at 37°C. The FITC-conjugated Annexin V positive cell and intracellular DOX localization was visualized under a fluorescence microscope (Nikon Eclipse 80i, Nikon, Japan). Representative images shown were obtained using a fluorescence microscope Eclipse 80i (Nikon, Japan) (Plan Fluor, 40X, NA 0.75 objective) equipped with green and red filters for FITC and DOX, respectively. (TIF) [file pone.0094309.s004.tif]

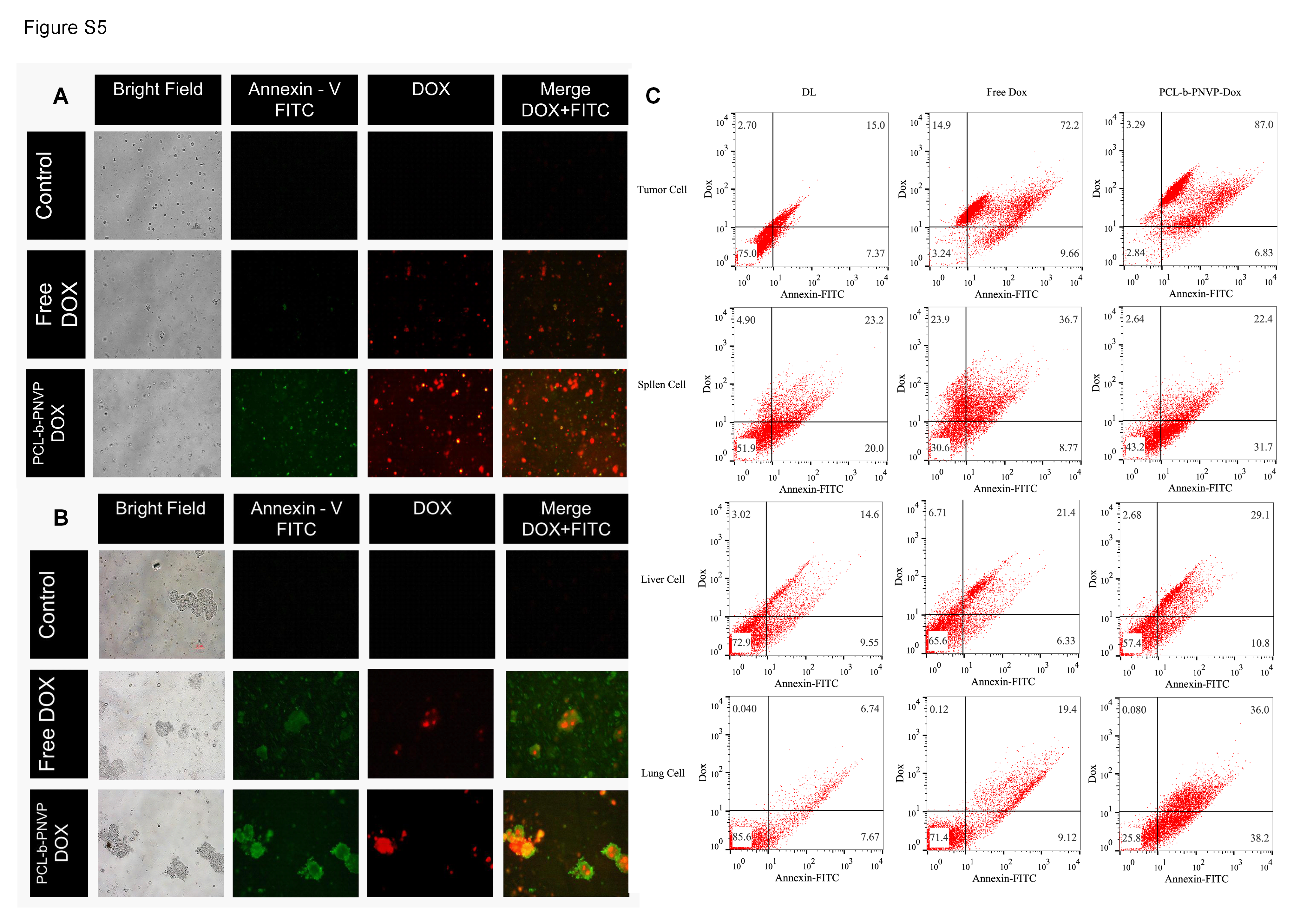

Supplement: Figure S5 — Apoptosis of tumor cells in targeted organs upon treatment with DOX- PCL63-b-PNVP90. DOX-PCL63-b-PNVP90 micelle induces apoptosis in tumor cells metastasize in spleen (A) and liver (B) as judged by microscopic analysis. FACS analysis of Annexin V positive cells with doxorubicin uptake in tumor cell alone and tumor cells metastasize in spleen, liver and lung of mice treated with either free DOX or, PCL63-b-PNVP90 micelles (C), n = 3. (TIF) [file pone.0094309.s005.tif]

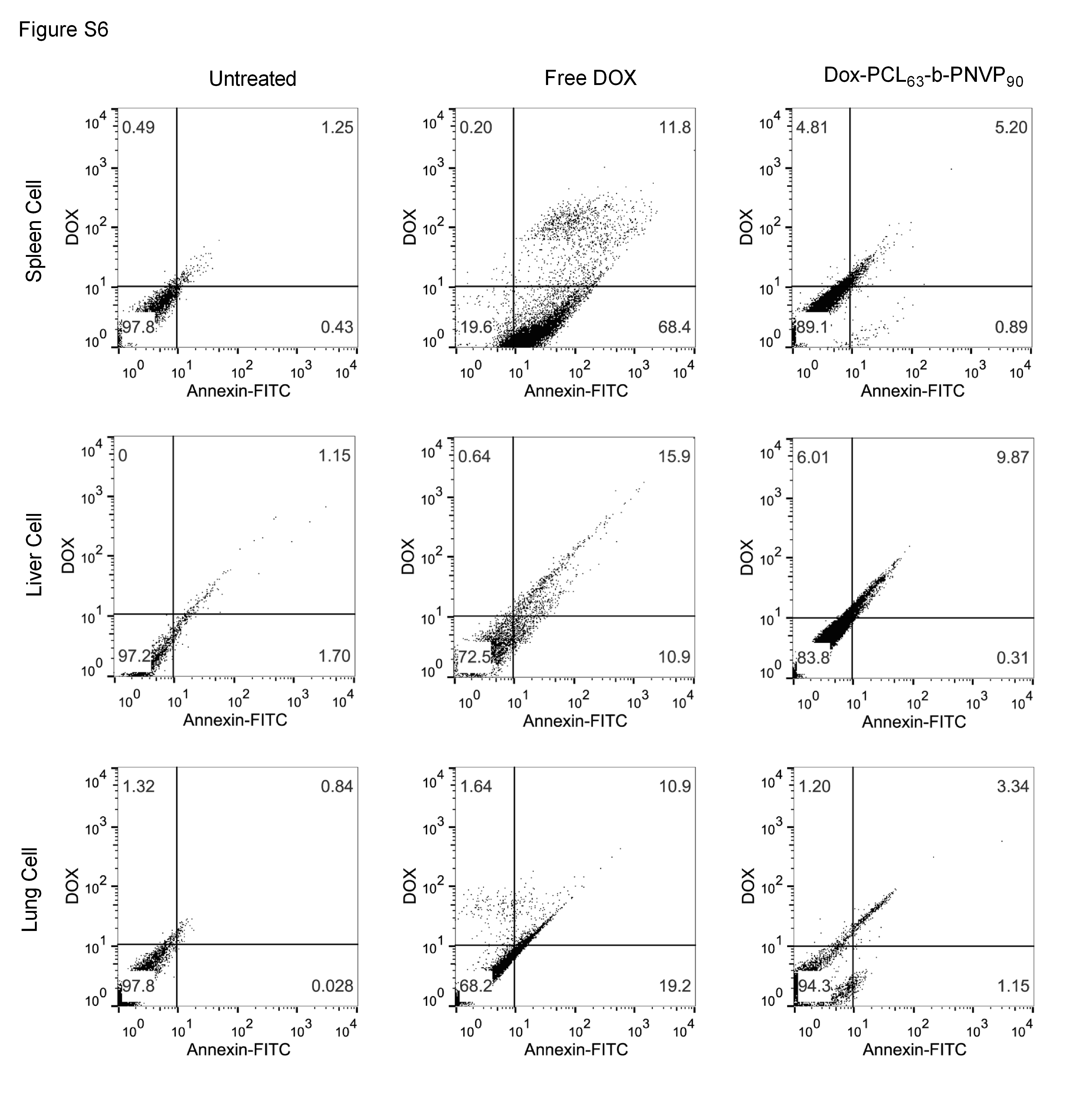

Supplement: Figure S6 — Apoptosis of normal liver, spleen & lung cells upon treatment with DOX or DOX-PCL63-b-PNVP90. Spleen, liver & lung cell apoptosis was assessed by FACS analysis of Annexin V positive cells following doxorubicin uptake. Normal mice (4 per group) were treated with either free DOX or, DOX-PCL63-b-PNVP90 micelles and were kept for 22 days. The mice were sacrificed after 22 days and spleen and liver cells were harvested. The cells were stained with Annexin V-FITC and analysed in FACS calibur as performed above. At least 10,000 cells were counted with triplicate determination. (TIF) [file pone.0094309.s006.tif]
